# Dead Matter, Living Machines: Repurposing Crustaceans' Abdomen Exoskeleton for Bio‐Hybrid Robots

**DOI:** 10.1002/advs.202517712

**Published:** 2025-11-26

**Authors:** Sareum Kim, Kieran Gilday, Josie Hughes

**Affiliations:** ^1^ The CREATE lab Institute of Mechanical Engineering School of Engineering, EPFl Lausanne 1015 Switzerland

**Keywords:** bio‐hybrid robot, bio‐joint, biotic material, cyclic design, sustainable material

## Abstract

Bio‐hybrid robots utilize living organisms for robot design, however, their use of living bodies makes maintenance, control, and fabrication of robot challenging. As an alternative, exoskeletons stand out for retaining mobility after the organism's death, making them an accessible candidate. In particular, crustacean exoskeletons, often discarded as food waste, provide both structural strength and flexibility from their segmented rigid shell. By repurposing dead animals' part from bio‐waste, a sustainable cyclic design process is proposed in which materials can be recycled and adapted for new tasks after a robot's lifespan. In this paper, a bio‐hybrid robot design using the langoustine abdominal exoskeleton as a bending actuator is introduced. Through integration with synthetic components, augmented exoskeletons can generate diverse, fast, and robust motions with extended operational lifetimes. Three robotic applications are demonstrated using a 3 g exoskeleton capable of supporting a 680 g payload: a manipulator handling objects up to 500 g, fingers that grasp various objects and bend at speeds up to 8 Hz, and a swimming robot at speeds up to 11 cm s^−1^. The method offers a sustainable robot design scheme and can be extended to diverse scales and functionalities by exploring a wide range of repurposable exoskeletons from bio‐waste.

## Graphical abstract

Crustacean exoskeletons, repurposed from food waste, are engineered into sustainable bending actuators combining biotic structure with synthetic control. The augmented exoskeletons achieve rapid and robust motion with lightweight body and can be used as part of robotic manipulators, grippers and swimmers. The approach establishes a recyclable bio‐hybrid design framework for sustainable and adaptable robotic systems.

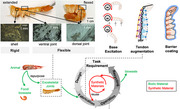

## 1 Introduction

Nature has long inspired robot design by demonstrating high performance and specialized actuators, sensors and mechanisms, setting challenging performance benchmarks for robots.^[^ ^1^ ^]^ Bio‐inspired robots often fall short of matching natural capabilities,^[^ ^2^, ^3^, ^4^, ^5^ ^]^ and one of the key reason is relying on synthetic components which are limited in terms of material properties,^[^ ^6^ ^]^ energy efficiency,^[^ ^7^ ^]^ energy storage,^[^ ^8^ ^]^ and adaptation.^[^ ^9^ ^]^ To overcome these limitations, bio‐hybrid robots combine living organisms with synthetic components.^[^ ^9^ ^]^ Actuation using animal tissues and cells has demonstrated greater efficiencies.^[^ ^10^, ^11^, ^12^ ^]^ However, the resulting robots remain fragile, requiring precise environmental control (e.g., of the surrounding nutrients, temperature, pH) and can need complex and expensive cell‐culturing setups. Plant‐hybrid robots have shown easier maintenance and deployment,^[^ ^13^, ^14^ ^]^ but inherently too slow and a lack of agility. Integration of robots with living animals to create cyborgs using neural controllers or mechanical stimulation can better leveraging the physical capabilities of animals,^[^ ^15^, ^16^, ^17^ ^]^ however, there are associated challenges in interfacing with species and significant ethical concerns.^[^ ^18^, ^19^ ^]^

To overcome the limitations of utilizing living organisms, *necrobotics* has been proposed, where the biological remains of dead organisms are integrated into robotic systems.^[^ ^20^ ^]^ This approach enables the exploitation of biological structures without requiring complex preservation of living matter, and eases the integration between synthetic and biological structures. Notable examples of this approach include the use of spider legs as robotic grippers,^[^ ^20^ ^]^ beetle exoskeletons for high‐load‐bearing structures,^[^ ^21^ ^]^ crawling robot with lizard skeleton^[^ ^22^ ^]^ for gait analysis, repurposing moth wings for odor sensing,^[^ ^23^, ^24^ ^]^ and using stimulate‐responsible fungi as a robot controller.^[^ ^25^ ^]^ However, existing necrobotic systems has functionally constrained due to their narrow design space, low‐force and low‐range actuation capability, and short operational lifespan, hindering their broader applicability in diverse robotic contexts.

**Figure 1:**
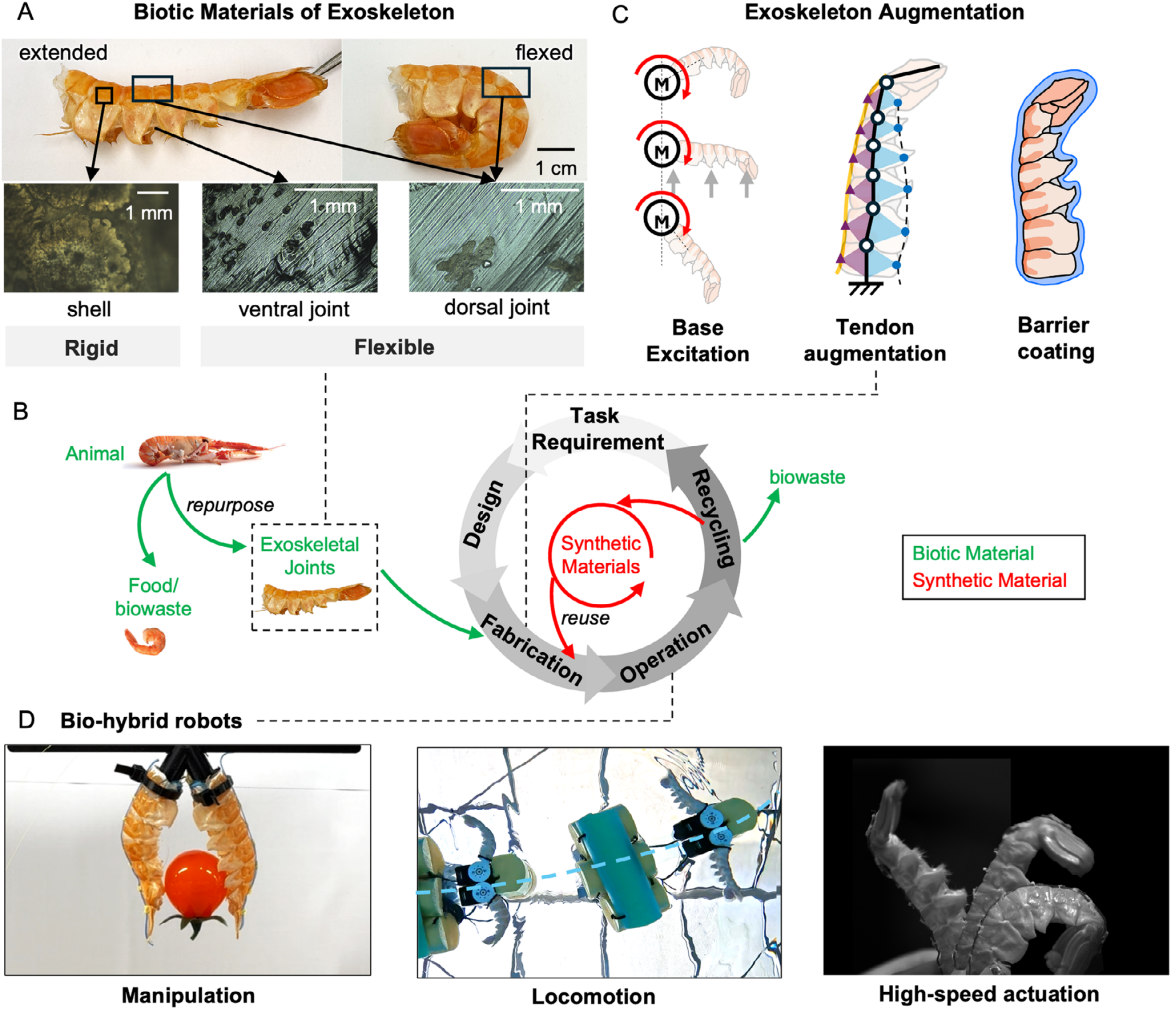
Repurposing Biotic Mechanisms for Robotics: A) Design process using repurposed joint from nature into robot design B) material properties of rigid and flexible components in the exoskeleton, each is a shell and a joint element, respectively C) design principles of bio‐hybrid robots incorporating passive actuation, active augmentation, and barrier coatings D) robotic applications including manipulators, locomotion systems, and high‐speed actuators.

To better leverage the advantages of biological structures from dead animals without being constrained by their original kinematics, we propose a bio‐hybrid design that utilizes exoskeletal bio‐joints. Exoskeletons are present in a wide range of animals, and include many different joint types existing at different scales. Exoskeletons combine rigid, mineralized shells with flexible joint membranes, providing both protection and mobility.^[^ ^26^, ^27^ ^]^ **Figure** **1**A shows an example of a langoustine abdominal exoskeleton. This balance of rigidity and flexibility allows independent segment motion for rapid, high‐torque motions for steering and maneuvering in water for crustaceans, such as during swimming^[^ ^28^ ^]^ or agile escape responses.^[^ ^29^, ^30^, ^31^ ^]^ These structures have been extensively studied^[^ ^32^, ^33^, ^34^ ^]^ and have inspired numerous robotic mechanisms, such as manipulators based on the joint fold design based on arm of a lobster,^[^ ^35^ ^]^ or the abdomen of lobster^[^ ^36^ ^]^ or a shrimp.^[^ ^37^ ^]^ Moreover, their structural integrity remains largely unaffected by biodegradation over practical timescales when compared to other soft biotic materials,^[^ ^38^, ^39^ ^]^ making them suitable for long‐term functional use.

With exploiting exoskeletons for bio‐hybrid robot use, we propose a circular design paradigm in which materials can be reused, recycled or biodegrade (Figure 1B). For a given task, the robot is designed, and is then fabricated by combining exoskeletal structures, which are biowaste and sometimes sourced as the waste byproduct of cooking process, with synthetic materials, namely actuators. To achieve a wide possible design space of possible kinematic motions with the exoskeletal structures we propose three different methods for augmenting their structures (Figure 1C). First, passive actuation leveraging their anisotropic properties through base actuation; second, active augmentation via tendon routing and elastomer embedding; and third post‐processing through coating to extend operational lifespan. These augmentations allow for the kinematic and dynamic properties of the exoskeleton to be altered for the specific task. After the operation, the exoskeleton and actuator unit can be separated, and the biodegradable joints naturally decompose, while most of the synthetic components can be reused, minimizing waste and enabling sustainable iteration.

In this paper, we demonstrate this design methodology using langoustine abdomens, lightweight and compact exoskeletal structures. These are particularly relevant due to their wide use in cooking, and hence wide availability as a waste product. Structurally, their exoskeletons can achieve large bending angles due to their segment rigid shells, which are connected by flexible joints at dorsal and ventral part as shown in Figure 1A. Using these exoskeletons, we show that through our design process and augmentation, we can develop robots for various robotic applications Figure 1D. Depending on the augmentation method, the robot can perform different tasks. This includes directional motion for swimming from passive actuation, and grasping and high‐speed bending up to 8 Hz from active actuation. This demonstrates how our approach not only facilitates the use of waste bio‐components but also leverages the advantageous features of the bio‐joints whilst expanding the design space of bio‐hybrid robots.

This study is the first example of integrating food waste into robotic systems, suggesting the sustainable robot design^[^ ^40^ ^]^ with reusing and recycling. Not just from the showcased langoustine joints, diverse types of joints exist in various scales can generate wider design space upon the desired task of the robot. For example, among decapod species, the 6‐joint abdominal exoskeleton has a structurally consistent design but varies in size and mechanical properties, providing room for scalable design. Therefore, other shellfish and exoskeleton systems, as well as further augmentation approaches, hold promising potential for future development.

## 2 Results

In this section, we demonstrate the range and characteristics of bio‐exoskeletal joints found in nature, followed by the selection of a joint from a modal animal. The selected joint guides the integration with synthetic materials to construct the robotic structure upon desired robot task. We showcase the joint augmentation methods and how these allow for diverse behaviors when interacting with the environment. Finally, we demonstrate a range of robotic applications, from manipulators to locomoting robots, highlighting the potential of the proposed approach to be augmented and further developed into functional robotic systems.

### 2.1 Exoskeletal Bio‐Joints in Nature

**Figure 2:**
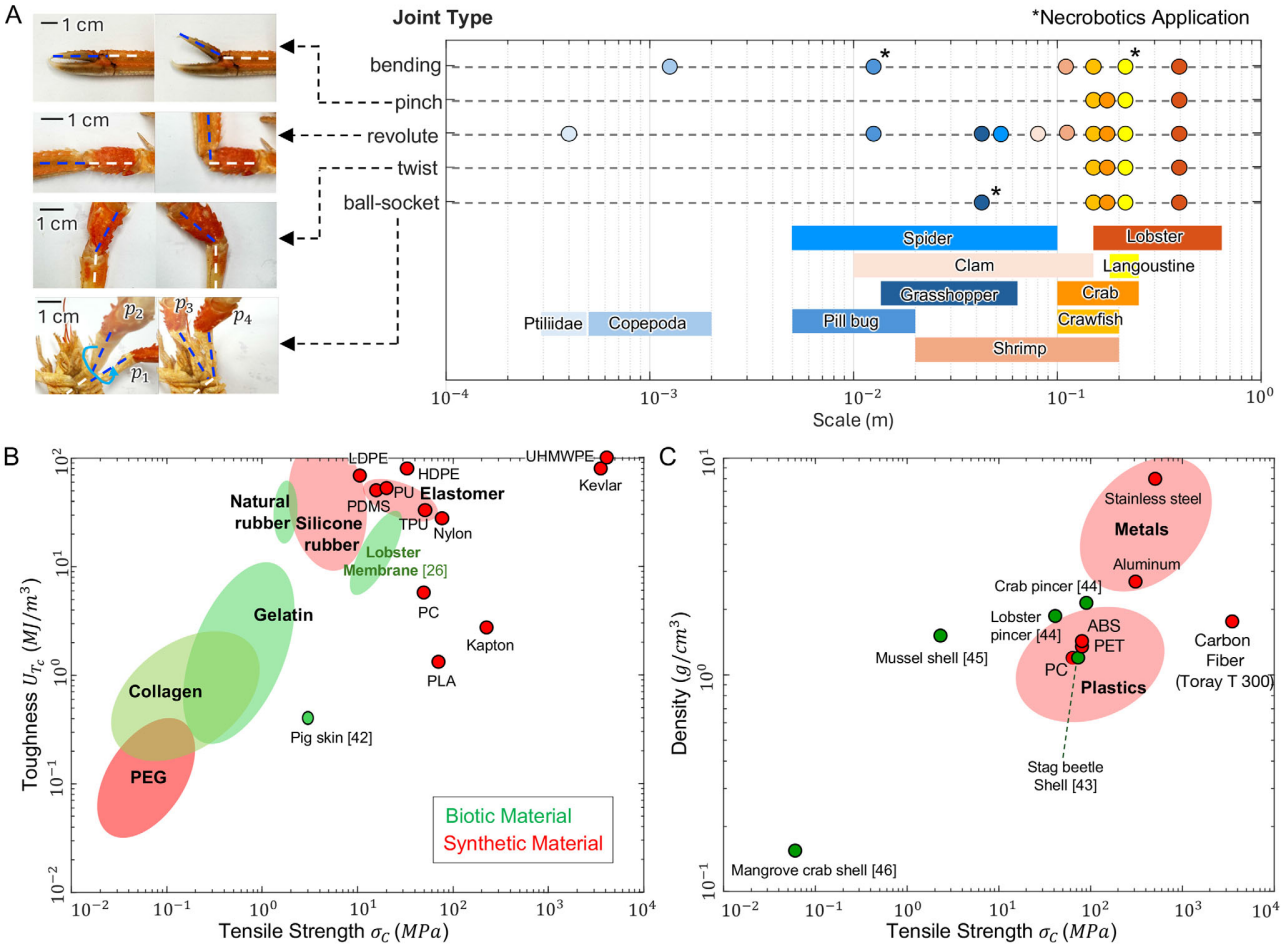
Diverse repurposable exoskeleton in nature. A) Various joint types across multiple scales and species of arthropods with an example of a langoustine joints, all scale bars represent 1 cm, B) material properties of the exoskeletal joints, and C) material properties of exoskeletal shell, both compared with other biotic and synthetic materials.

Nature offers diverse types of exoskeletal joints across species and scales providing favorable mechanical and material properties, as illustrated in **Figure** **2**. Figure 2A shows a variety of exoskeletal joint types commonly found in arthropods and their distribution across a range of animal sizes, from the 0.3–0.5 mm Ptiliidae to a 15–60 cm lobster in length, offering a wide range of design spaces for bio‐hybrid robotic systems. For example, langoustine, in addition to the abdomen bending joints introduced in Section 1, exoskeletons alone have four more different joint types. This includes pinch joints in the thumb claw, enabling high‐torque pinch‐grip used by many crustaceans for capturing prey or cutting.^[^ ^41^ ^]^ revolute joints with ≈90° of range of motion for arm flexing, twist joints enabling out‐of‐plane rotation in the arm, and ball‐and‐socket joints providing smooth rotating motion (*p*_1_ to *p*_4_ denotes timestamps in chronological order during 1 rotation) at the body–leg interface. The two linkages, indicated by blue and white dotted lines, show how their axes change according to the motion of the connecting joint. While arthropods predominantly exhibit revolute joints in their legs, certain insects (e.g., pill bugs^[^ ^17^ ^]^) and crustaceans (e.g., crawfish, langoustines, lobsters) possess ventral bending joints in their abdomen. In some larger insects and crustaceans, ball‐and‐socket joints are observed at the interface between the torso and the legs. As the body size of animals increases and organisms become more complex, the diversity of joint types tends to increase.

Figure 2B presents a comparative plot of tensile strength and toughness, defined as the amount of energy per unit volume that a material can absorb before failure, between exoskeleton joint membrane materials and synthetic joint materials. Biotic materials generally exhibit lower tensile strength and toughness compared to synthetic polymer materials commonly used in robotic joints, e.g., Ultra‐high‐molecular‐weight polyethylene (UHMWPE), Kevlar, thermoplastics (low‐density polyethylene (LDPE), high‐density polyethylene (HDPE), Polycarbonate (PC), polylactic acid (PLA)), elastomers (polyurethane (PU), thermoplastic polyurethane (TPU), polydimethylsiloxane(PDMS)), Nylon, and Kapton. The lobster joint membrane^[^ ^26^ ^]^ demonstrates notably good tensile strength and toughness relative to other biotic materials such as natural rubber, gelatin, collagen (data collected from ref. [26]), and animal skin from pig.^[^ ^42^ ^]^ Although it shows weaker tensile strength than synthetic materials, it possesses high toughness comparable to natural rubber.

Figure 2C presents a comparative plot of density and tensile strength for shell materials including both biotic and synthetic materials. While biotic shell materials are lighter than commonly used synthetic materials such as metals(aluminum and stainless steel), they demonstrate varying mechanical properties. The stag beetle shell^[^ ^43^ ^]^ offers comparable strength to plastics such as PC, polyethylene terephthalate (PET), acrylonitrile butadiene styrene (ABS), with lower density. The highly mineralized and structurally ordered chitin found in lobsters' and crabs' pincers^[^ ^44^ ^]^ shows similar or slightly lower tensile strength than plastics (PC, PET, ABS). The mussel shell^[^ ^45^ ^]^ exhibits significantly lower density than carbon fiber but demonstrates relatively modest tensile strength. In contrast, the mangrove horseshoe crab^[^ ^46^ ^]^ exhibits a less calcified, more fibrous chitin structure that prioritizes flexibility over strength, resulting in both lower density and reduced tensile strength, highlighting the diverse mechanical strategies evolved by biological systems to meet specific functional requirements.

Based on these observations, as a modal exoskeleton, we selected the bending joint of the langoustine abdomen. This selection is driven by several key factors: its moderate size, high mechanical strength from mineralized and structurally ordered chitin and its large bending angles from tough yet thin joint membranes, resulting in a robust yet flexible joint, having a potential for diverse robotic applications.

### 2.2 Characteristics of Abdomen Exoskeleton

Langoustine (*Nephrops norvegicus*), commonly known as Norway lobster, are small, slender crustacean belonging to the decapod order of the phylum Arthropoda, closely related to shrimps, lobsters, and crayfish. Their exoskeletons are composed of thick, mineralized plates that provide high stiffness and hardness^[^ ^33^ ^]^ offering protective function,^[^ ^47^ ^]^ yet segmentation interconnected by flexible membranes, provides mobility.^[^ ^26^, ^48^ ^]^ In many decapods, including langoustines, the ventral section comprises six articulated segments forming the abdomen.^[^ ^34^ ^]^ This stiff yet flexible structure enables rapid, complex motions primarily in the dorso‐ventral plane. Its structural adaptability highlights the evolutionary balance between protection and maneuverability in arthropod exoskeletons. In this study, we first characterize and analyze this exoskeletal mechanism to inform the design of bio‐hybrid bending actuators for robots.

**Figure 3:**
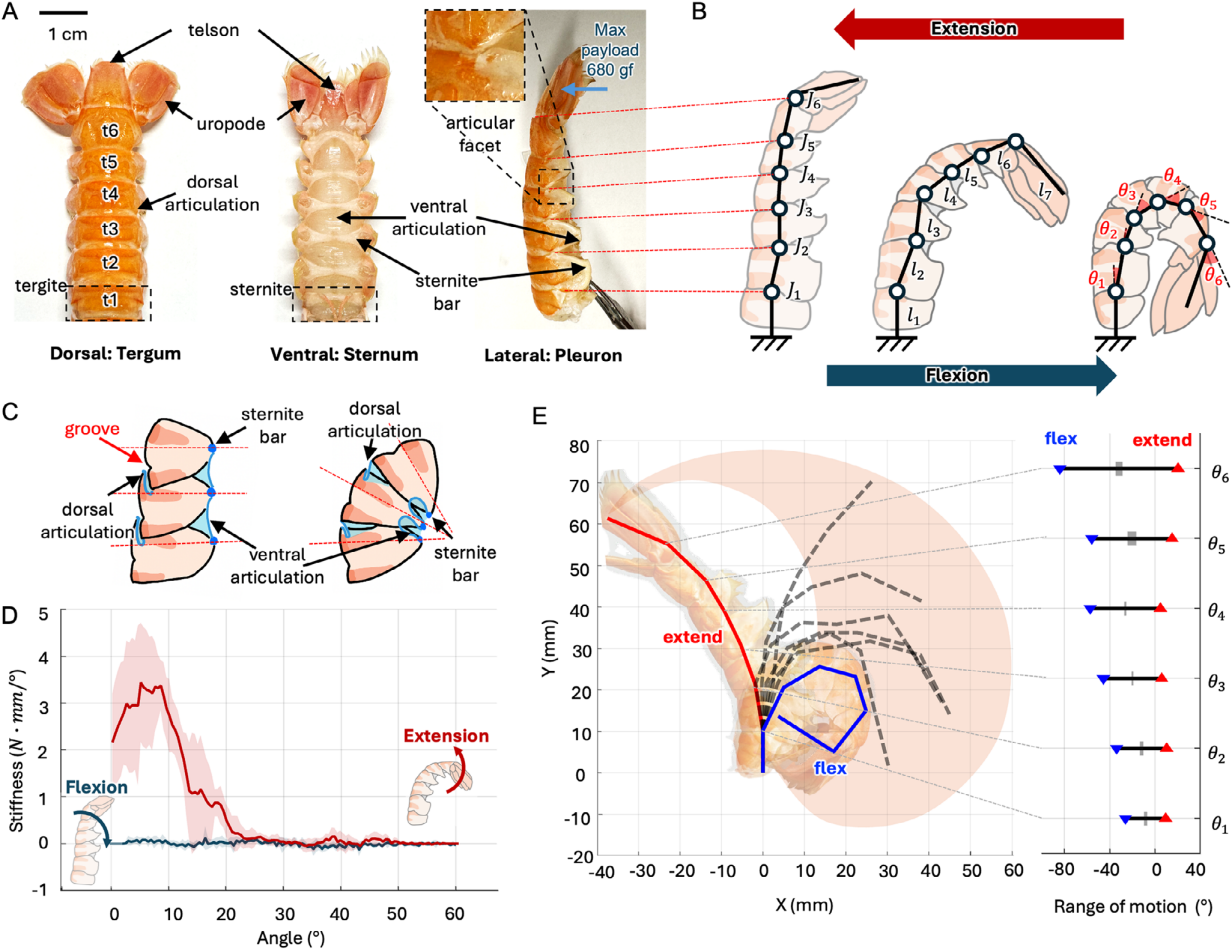
Characterization of the natural abdomen exoskeleton in langoustine. A) Anatomy and segmentation of the exoskeleton shown from dorsal, ventral, and lateral views B) extension and flexion postures of the abdomen and associated linkage parameters C) schematic of the joint mechanism illustrating the groove‐guided angle constraint D) anisotropic stiffness response during extension and flexion E) workspace and range of motion of the joints, visualizing the angular limits and diverse possible configurations of exoskeleton.

**Figure** **3**A illustrates the structure of the abdomen of langoustine,^[^ ^49^ ^]^ a continuum mechanism composed of six segmented unit called somites (labeled T1 to T6), each encased by distinct exoskeletal components: the dorsal segment (tergite), and the lateral segment (pleurite). The tergite forms a rigid shell, providing structural protection, and is connected to adjacent tergites via dorsal articulations. The ventral portion, composed of relatively softer sternites, contains ventral articulations and is often reinforced by a sternite bar enhancing mechanical stability. Each somite is linked to its neighbors through these articulations, which form an articular facet—a smooth, cuticular contact surface between adjacent exoskeletal elements forms the interface for articulation in lateral view (inset shows magnified detail). Although this facet has been described as a ball‐and‐socket joint,^[^ ^49^ ^]^ functionally it behaves like a revolute joint with constrained rotation.

This complex arrangement of rigid and flexible elements reflects a functional balance: rigid components, such as the tergite and sternite bar—composed of calcified chitin—provides mechanical strength and protection, while the articulations—made of non‐calcified chitin—allow flexibility. These articulation membranes are tough, inextensible, and resistant to biodegradation. The integration of dorsal and ventral articulations enables the abdomen to perform both rapid, forceful curling for escape responses and finer, controlled movements necessary for swimming and burrowing. At the posterior end, the abdomen terminates in the flat plate segment (telson), flanked by paired appendages (uropods) that together form a tail fan for propulsion and maneuverability.

Figure 3B illustrates schematic representations of the abdomen in extension and flexion, along with a simplified model of the abdominal joints. In this model, the articulation facet functions as a revolute joint (J1 to J6), and each somite is modeled as a rigid linkage with length *l*_1_ to *l*_6_. The corresponding joint angles are denoted as θ_1_ to θ_6_, are each limited to a fixed range of motion determined by the anatomical articulation and the geometric constraints of the exoskeletal structure. Figure 3C illustrates the biomechanical mechanism of abdominal extension (left) and flexion (right), highlighting deformation at the articulation joints in each state and explaining the factors that constrain joint angles. Each tergite features a dorsal groove and is connected to adjacent tergites through a dorsal articulation. During flexion, the grooved tergites roll over one another,^[^ ^36^, ^37^ ^]^ initiating rotation at the dorsal articulation. As flexion progresses, the dorsal joint continues to roll until it reaches its mechanical limit—marking maximum flexion—while the ventral articulation folds inward. In contrast, during extension, the dorsal components roll in the opposite direction. When the posterior edge of a tergite contacts the groove of its anterior neighbor, the system reaches maximum extension, at which point the ventral articulation is fully stretched.

The repurposed abdomen exoskeleton is an empty shell with jointed mechanism without mobility. The first key feature observed is its geometric anisotropy in stiffness due to asymmetric angular constraints. As shown in Figure 3D, the joint configuration is quantified by the opening angle of the abdomen, defined with respect to its maximum extension (θ_open_ = 0°) and increasing as it flexes up to θ_open_ = 60°. During flexion(θ_open_ is increasing from 0° to 60°, marked as blue line), the absence of such angular locking allows free deformation with low stiffness, resulting in a frictionless, compliant movement with minimal load transmission. In contrast, when the abdomen extends (θ_open_ is decreasing from 60° to 0°, marked as red line), the joints approach hard geometric limits, locking the structure and producing a sharp increase in stiffness (in between θ_open_ = 10° and 20°). The stiffness is increasing up to 3.3 N·mm/°, enabling the transmission and resistance of large external loads. The maximum payload of the abdomen until failure is 6.7 N (680 gf) observed in this ‘high stiffness’ configuration out of 3 g of exoskeleton. This directional stiffness asymmetry underlies the abdomen's ability to combine high load‐bearing capacity in extension with mechanical compliance in flexion, and can be a useful characteristic for the bio‐hybrid bending actuator design.

The second key characteristic of the exoskeleton is joint mobility and overall workspace, illustrated in Figure 3E. Each joint exhibited a distinct range of motion (RoM), with a trend of increasing bending capacity toward the posterior end (from somite T1 to telson direction). The maximum angles for six abdominal joints, J1 to J6 were ≈10, 10, 6, 5, 15, and 21 degrees (denoted as red triangle markers), respectively, while the minimum angles were –26, –34, –46, –57, –56, and –84 degrees (denoted as blue triangle markers). Standard deviations ranged from 2 to 8 degrees, indicating consistent yet slightly variable flexibility among individuals. In Figure 3E, the workspace of the manipulator is illustrated as an orange‐colored zone, with several potential abdomen configurations are drawn with dashed lines. These configurations correspond to different combinations of joint angles, with the flex limit represented where all joint angles are at their smallest (flex state), and the extend limit indicated by the red line, where the joint angles are at their largest (extend state).

These two characteristics demonstrate that the integrated abdominal structure combines flexible bending motion with high mechanical strength and anisotropic stiffness. These results demonstrate its remarkable combination of stiffness and flexibility, making it an ideal template for designing bio‐hybrid bending actuators for robot design.

### 2.3 Base Excitation for Generating Directional Forces via Structural Anisotropy

The structural properties of the exoskeletons can be leveraged for the structures of bio‐hybrid robotic mechanism. The first method is base excitation, which leverages the abdomen's anisotropy to generate directional force output for propulsion or object manipulation. As explained in Figure 3D, the abdominal exoskeleton exhibits an asymmetric force response due to its anisotropic stiffness during flexion and extension. This directional difference in mechanical response facilitates a simple yet effective actuation strategy: movement in one direction is easier than in the opposite, enabling repetitive motion to produce net force with minimal control effort comes from functional characteristics of the natural structure.

**Figure 4:**
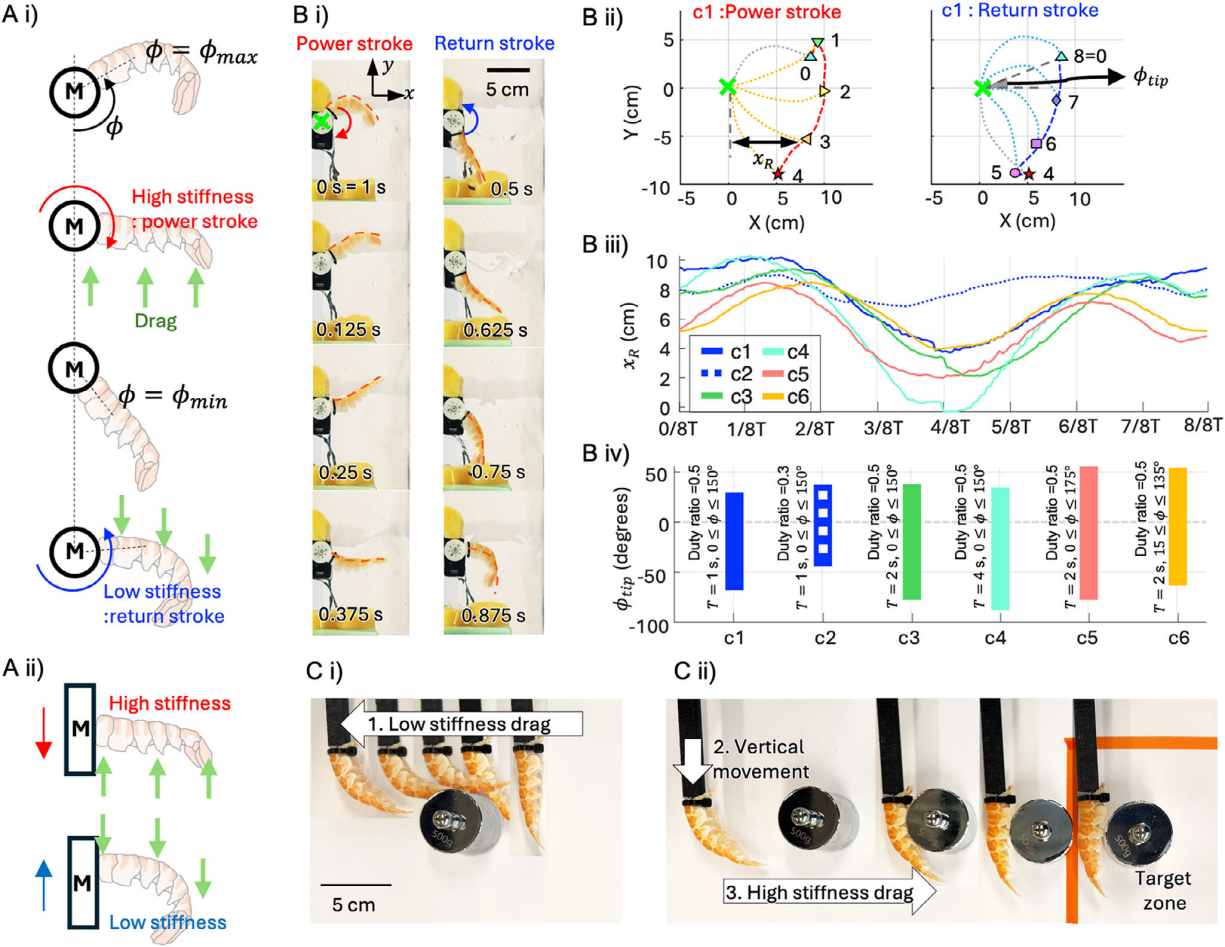
Base excitation of exoskeleton. A) Motorized stroke generation and stiffness variation upon moving direction of i) rotary motion and ii) linear motion B) underwater fin trajectory upon in a single flapping period i) snapshots during power and return stroke ii) fin profiles and performance parameters of projected width (*x*_*R*_) of drag and tip angle (ϕ_tip_), trajectory of fin upon different flapping parameters resulting different iii) projected width and iv) range of tip angle C) underactuated robot finger with anisotropic stiffness in different dragging direction, i) low stiffness drag and ii) high stiffness drag.

As shown in **Figure** **4**A, the exoskeleton is mounted on a base that is cyclically driven along either a circular or linear trajectory in a resistive medium, where the object experiences a drag force. In the circular trajectory (Figure 4A(i)), the exoskeleton is aligned with the motor (denoted as M) rotation axis. As the motor rotates, the opening angle ϕ varies between ϕ_max_ and ϕ_min_. When the motor rotates counterclockwise (decreasing ϕ), drag induces extension of the exoskeleton, increasing its stiffness and producing high resistance to the medium, thereby generating a power stroke. When the motor reverses rotation, the drag direction flips, inducing flexion of the exoskeleton. In this configuration, the exoskeleton exhibits low stiffness and minimal resistance, enabling the return stroke. Linear excitation (Figure 4A (ii)) operates similarly: motion in one direction (downward) produces high resistive force, while motion in the opposite direction (upward) produces low resistive force.

This passive exploitation of geometric locking allows effective actuation with minimal control complexity. Over a full actuation period, the asymmetry produces net thrust, effectively converting symmetric base motion into unidirectional propulsion. Figure 4B(i) and Movie S1 (Supporting Information) show that the exoskeleton's trajectory in water during the power and return strokes with a symmetric controller c1. The key control parameters include the period *T*, the duty ratio (defined as duration of power stroke/(duration of power stroke + return stroke)), and the flapping amplitude (defined by the angular range between ϕ_min _ and ϕ_max _). The controller c1 uses parameters *T* = 1, duty ratio = 0.5, and an angular range of 0° ⩽ ϕ ⩽ 150°. The corresponding configuration and trajectory of the exoskeleton are illustrated in Figure 4B(ii), with the power stroke shown on the left and the return stroke on the right. The full motion cycle is divided into eight frames. During the power stroke (frames 0–4), the fin transitions from return to power stroke between 0/8*T* and 1/8*T*, where the exoskeleton shifts from a flexed to an extended configuration. From 1/8*T* to 1/4*T* In the return stroke, from 4/8*T* to 5/8*T*, the system transitions from the power to the return stroke, changing from an extended to a flexed configuration. Between 5/8*T* and 8/8*T* (equivalent to 0/8*T*), the flexed exoskeleton continues sweeping to reach the maximum motor angle ϕ_max_. The trajectory and stiffness profiles differ between the power and return strokes, leading to an asymmetric cycle that generates net thrust. From the trajectory, we define two key parameters for quantitative comparison: the angular range of the exoskeleton tip, ϕ_tip_, and the projected width *x*_*R*_.

Changing the speed profile and degree of asymmetry of the controller can significantly change the motion of the exoskeleton‐structure. To explore this six different controllers (c1–c6) have been designed (paremeters are in the Table 1), each of which exhibit distinct trajectories. The duty ratio is adjusted by c1 and c2; the period *T* is modulated by controllers c1, c3, and c4; and the excitation angles are varied by c3, c5, and c6. More detailed information on the controller is provided in Section 4.

According to each controller, the projected width (*x*_*R*_) and range of tip angle (ϕ_tip_ are given for one actuation cycle in Figure 4B(iii,iv) respectively. Controller c1 uses *T* = 1 s with a duty ratio of 0.5, while c2 uses the same period with a lower duty ratio of 0.3. The reduced duty ratio in c2 results in a faster closing motion of the exoskeleton but yields a smaller difference in *x*_*R*_ between the power and return strokes. In comparison, c1 shows both a larger maximum and smaller minimum *x*_*R*_, indicating stronger asymmetry. Additionally, the opening angle range is narrower in c1 (–68° to 29°) than in c2 (–43° to 37°). Although faster flapping generally produces higher thrust,^[^ ^50^ ^]^ the effects of *x*_*R*_ asymmetry and angular range may compensate each other.

To examine the effect of flapping speed, controllers c1 (*T* = 1 s), c3 (*T* = 2 s), and c4 (*T* = 4 s) are compared while keeping the duty ratio at 0.5. As the period increases, both the difference in *x*_*R*_ and the angular range ϕ_tip_ become larger. However, these increases are balanced by the reduced thrust typically associated with slower power strokes. Controllers c3, c5, and c6 all use *T* = 2 s and a duty ratio of 0.5 but differ in their flapping angle ranges. Controller c5 operates with a wider range (0° ⩽ ϕ ⩽ 175°) than c3 (0° ⩽ ϕ ⩽ 150°), while c6 uses a narrower range (15° ⩽ ϕ ⩽ 135°). Among these, c5 shows the largest ϕ_tip_ range and the greatest difference in *x*_*R*_, suggesting a strong correlation between angular amplitude and stroke asymmetry.

While this analysis does not directly measure thrust, since drag‐based thrust is a complex behavior depending on both flapping speed and resistive area, it provides insight into how different control parameters affect thrust generation. Variations in temporal and angular parameters produce distinct kinematic patterns, as reflected in the projected width and angular displacement. Through cyclic rotary motion, the resulting asymmetric behavior functions like a swimming fin, generating net thrust for propulsion. This can be exploited to enable swimming robots, and is explored later in Section 2.5.

Asymmetric force output can also enable the exoskeleton to be used as a passive manipulator to handle an object without complex control, as illustrated in Figure 4C and in Movie S2 (Supporting Information). When the exoskeleton interacts with a 500 g object during a low‐stiffness drag, it passively flexes and generates a small reaction force, insufficient to move the object. After a small vertical adjustment that aligns the abdomen exoskeleton with the object, dragging in the opposite direction causes the exoskeleton to stiffen and generate a high force, successfully pulling the object toward the target zone. This behavior is advantageous for manipulating and navigating confined spaces, as the manipulator can pass through without complex control by conforming its shape to obstacles, while modulating its force output simply by changing the dragging direction.

### 2.4 Tendon Augmentation for Complex Abdomen Morphology

Through base excitation, the abdomen exoskeleton can function as a simple bio‐hybrid actuator when combined with simple cyclic motor actuation, leveraging its inherent mechanical properties. However, the behavior of exoskeleton is highly dependent on the environment and remains passive. To achieve precise control and generate diverse motions, optimized for specific robotic tasks and high‐performance, we propose *augmenting* the abdominal exoskeleton with tendons and elastic elements.

#### 2.4.1 Augmented Exoskeleton Design with Tendon‐Driven Actuation

The goal of tendon augmentation is to maintain the underlying advantageous properties whilst increasing the controllability and task specificity. Unlike the living form, where abdominal motion is driven by muscle sets, the inanimate exoskeleton has a hollow continuum shell, requiring a compatible actuation strategy. Tendon actuation is well suited as it can be routed along the natural curvature, transmit high force, and replicate muscle‐like distributed deformation while maintaining the lightweight structure.

**Figure 5:**
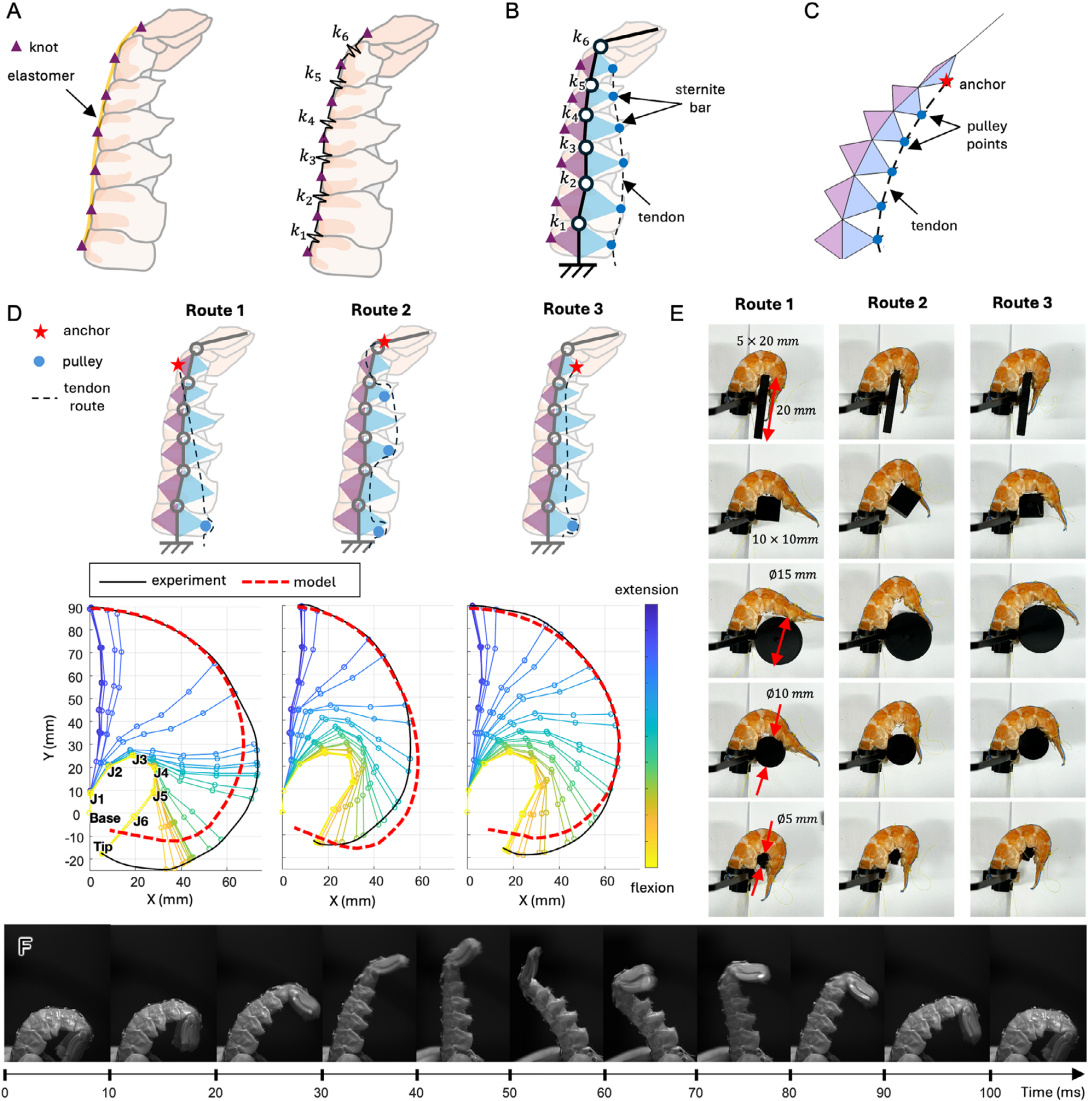
Tendon‐augmented abdomen exoskeleton. A) Embedding of tendons within an elastomer and its equivalent mechanical model B) kite‐shaped approximation of each somite, showing potential tendon fixation points and an example routing configuration C) simulation of tendon pulling based on the simplified model D) experimental results showing the tip trajectory under different tendon routing configurations E) configurations of the augmented exoskeleton during interaction with objects of varying geometries F) high‐speed actuation of the augmented exoskeleton at 8 Hz.

Since tendons can only exert tensile force (pulling), achieving antagonistic actuation for flexion and extension typically requires two tendons and actuators, increasing system complexity. To simplify this, we introduce an elastomer as a restoring element. Mounted along the centerline of the dorsal shell, the elastomer is arranged in tension when the structure is in its maximum extended state. By creating an energetic gradient upon flexion, this configuration guides deformation and recovery, and provides a restoring force that returns the structure to extension after tendon‐driven flexion (**Figure** **5**A).

When the elastomers are tied at the center of each tergite with a knot, each segment between the tergites acts independently. Kinematically, the system can be modeled as a series of spring elements between rigid linkages (Figure 5A), with spring constant *k*_1_ to *k*_6_, respectively. This configuration enables antagonistic motion using a single tendon, while the fast recovery of the elastomer allows for high‐speed actuation. The actuating tendon is embedded in the ventral part of the shell (Figure 5B) and passes through the stiff sternite bar, which acts like a rigid pulley. When the tendon is pulled, the abdomen flexes.

Since the exoskeleton has 6 DoF, a single tendon drive mechanism is underactuated. To explore the design space of underactuated exoskeleton bending and predict motions for different tendon routings, we developed a kinematic model that estimates the exoskeleton's pose based on tendon routing and pulling. In each segment, the elastomer fixation point (purple triangle), the rotational joints connecting the linkages (white circles), and the tendon pulley points (blue circles) form an approximately kite‐shaped structure. A schematic of this simplified kinematic chain is shown in Figure 5C.

By changing the routing of the tendon, the flexing motion of the joint can be modulated. Figure 5D shows three different tendon routings, and corresponding trajectories. Here, the anchor point is the fixed end of the tendon where the path is started. The pulley points are potential locations where the tendon passes over pulleys and changes direction — in other words, points that guide the tendon along its path. For example, route 1 has its anchor point at the dorsal side of the sixth link, and pulley point over the ventral sternite of first link. The result trajectories highlight how a diverse range of motion of the abdomens can be achieved by varying the tendon routing. Route 1 has smooth bending from the base to the tail, and rapid snap of the last joint at the last step. Route 2, first, third and fifth ventral segments are the pulley points, and the anchoring point is on the edge of the very last linkage. In route 2, joint 4 bends first and is followed by the other joints. This shows a narrower workspace than the route 1 joint configuration. Route 3 has a pulley point on first segment ventral section, and its anchoring point on the sixth ventral segment. It is characterized as having smooth, continuous bending from base to tail. The experimental results (black‐colored solid line) and kinematic model results (red‐colored dotted line) show a good match.

Due to the structure's flexibility and underactuation, the abdominal exoskeleton's behavior can vary when interacting with other objects. Figure 5E shows diverse grasping configurations of the abdominal exoskeleton attempting to grasp objects of various sizes and shapes (a thin plate, a small cube, and cylinders with three different diameters), illustrating how the exoskeleton conforms to each object. In tendon routing 1 and 3, bending starts from the distal end, resulting in high conformity to external objects, which leads to larger contact areas and smaller gaps during grasping. Compared to routing 3, routing 1 tends to exhibit a snap‐through motion in the tail‐end segments when approaching the flex limit, which reduces conformity for larger objects.

Due to this tendon‐driven mechanism with single degrees of freedom actuation, rapid actuation is possible through an antagonistic mode in which recovery force drives motion in one direction. As shown in Figure 5F and Movie S3 (Supporting Information), the abdominal exoskeleton can be actuated at speeds of up to 8 Hz, where extension takes 40 ms and flexion 60 ms. This fast speed of actuation is comparable to the escape motion speed of a crayfish.^[^ ^31^ ^]^

#### 2.4.2 Barrier Coating

**Figure 6:**
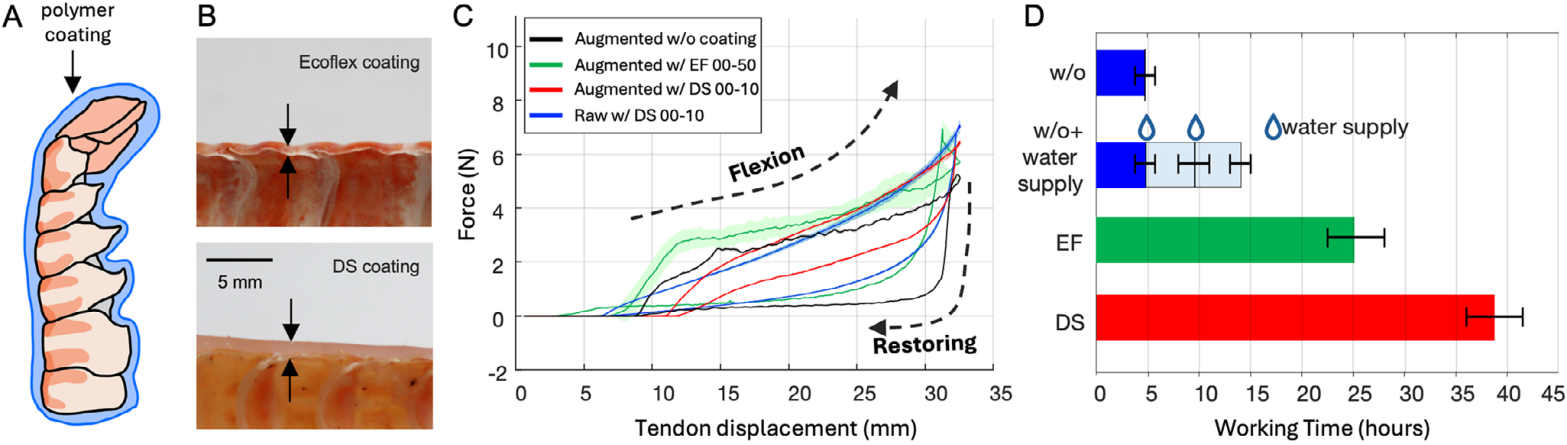
Barrier coating. A) Schematic of abdomen with polymer coating layer, B) cross section of the barrier coating with ecoflex (EF) and dragonskin(DS) on the dorsal exoskeleton, C) hysteresis curves obtained from 5 repeated flexion‐restoring cycles driven by tendon actuation under four coating conditions: augmented w/o coating, augmented with EF, augmented with DS, and raw with DS coating. The lines show mean values, and the shaded areas represent standard deviations, D) working hour change depends on different coatings, evaluated on four samples per coating condition.

The exoskeletons have evolved to operate in an aquatic environment, relying on the hydration for joint lubrication and preservation of the material properties. The joint material is uncalcified chitin,^[^ ^51^ ^]^ which is soft and flexible when it contains 90% of moisture,^[^ ^52^ ^]^ but turns stiff and hard when it dries out. To maintain actuator performance, we sealed the abdomen exoskeleton with a barrier coating. We used dip coating of the silicon, which can form a thin, uniform protective layer on the abdomen, as shown in **Figure** **6**A, to ensure minimal effect on the underlying range of motion or stiffness of the joint. Two different coating materials–Ecoflex(EF), DragonSkin(DS) with different viscosities were tested, which in turn lead to different thickness (≈200 µm with EF coating, and ≈1 mm with DS coating) as shown in the cross‐sectional image in Figure 6B. The silicones do not adhere to the exoskeleton or chitin‐based materials.^[^ ^32^ ^]^ However, once cured, the coating remains as a continuous, mechanically constrained sleeve over the exoskeleton. This is advantageous as this sleeve can move over the surface when structure is actuated, so it remains intact as a continuous barrier.

Figure 6C shows force profiles across five tendon actuation cycles for each sample. During tendon pulling, the force increases throughout flexion, and during restoring (extension), the force drops sharply once the tendon begins to release. This results in a large hysteresis in the cycle. Compared to the sample without (w/o) coating, the addition of a coating reduces hysteresis due to the stiffness of the polymer layers. The maximum force is higher with the DS coating than with the EF coating. Comparing DS coating with and without the elastomer, the force profile is smoother with the elastomer, while the peak force remains similar—because the dominant restoring force comes from the stiffness of the DS layer. Overall, EF coating showed larger variability across repeated cycles with high standard deviation, whereas others exhibited good repeatability with small standard deviation.

To evaluate durability, the working time of the augmented joint was measured on four samples per coating condition. Without any coating, the augmented joint lasted for 4.8 h with a standard deviation of 0.8 h. When dipped in water for 10 min after the end of working hours, the working time is extended to 4.8 h with a 1.3‐h standard deviation after the first water supply, and 4.5 h with a 0.9‐h standard deviation after the second water supply, resulting in a total average working time of 14.1 h. This suggests that periodic water supplementation can further prolong joint durability. With the EF coating, the joint lasted for 25.3 h with a 2.4‐h standard deviation, which is more than three times longer than the uncoated case. The DS coating improved durability even further, extending the joint's working time to 38.8 h with a 2.4‐h standard deviation—about six times longer than without coating.

### 2.5 Robot Integration and Applications

**Figure 7:**
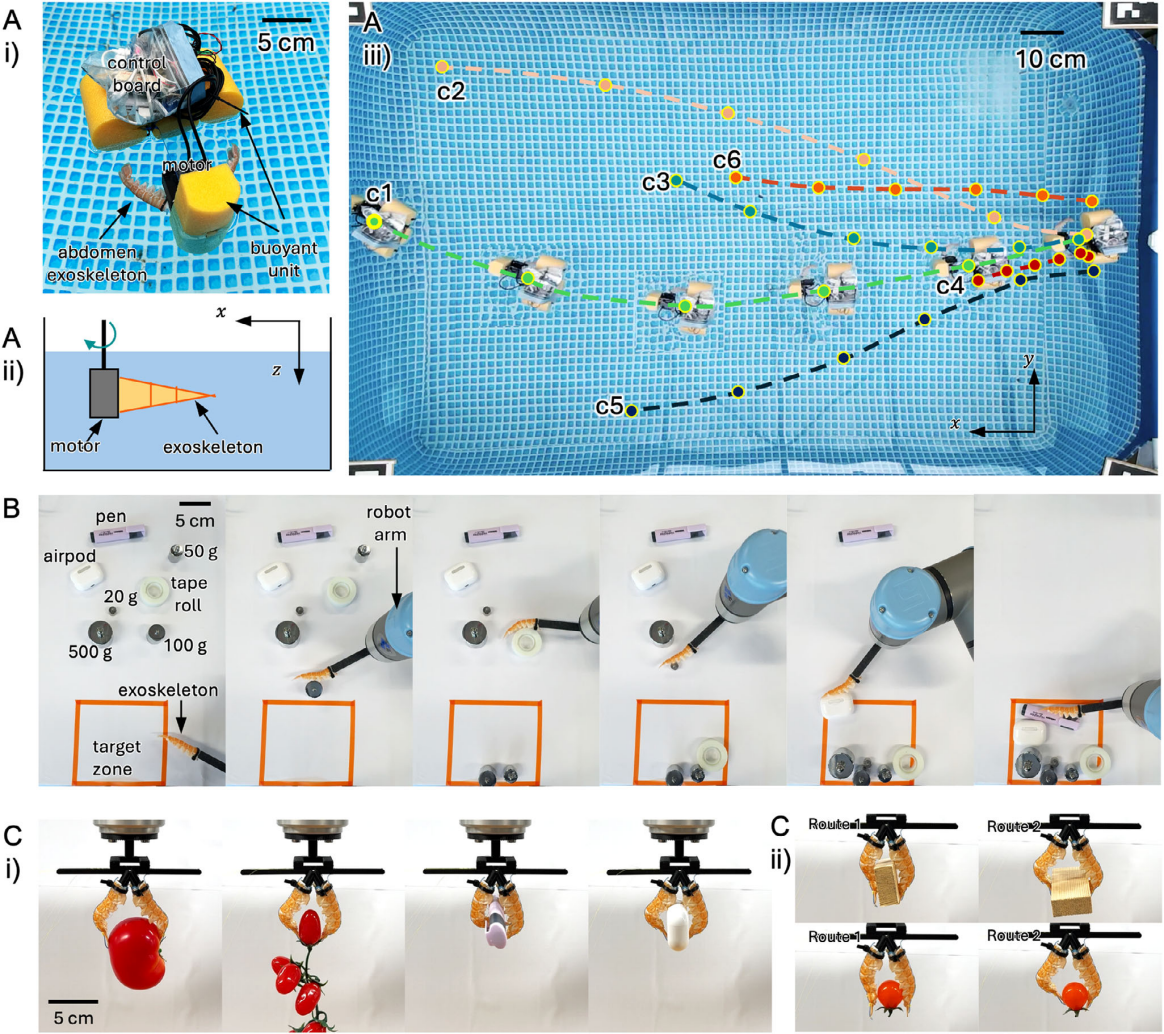
Robotic applications of the bio‐hybrid actuator. A) Swimming robot platform i) robot hardware with two flapping abdomen exoskeletons ii) flapping direction and fin orientation iii) robot trajectories upon diverse flapping commands from 1 to 6. B) object handling to relocate diverse items to target region C) two‐finger gripper with tendon‐driving augmented exoskeletons i) gripper grasping different objects ii) gripper configuration upon different tendon routes.

Building upon the design principles outlined in Sections 2.3 and 2.4, we developed bio‐hybrid robots which integrate the langoustine exoskeleton with synthetic components, as shown in **Figure** **7** with diverse tasks from locomotion to manipulation. First, with the base actuation method introduced in Section 2.3, an untethered swimming robot with two flapping exoskeletal fins is illustrated in Figure 7A and Movie S1 (Supporting Information). Two abdominal exoskeleton fins are symmetrically arranged on the robot (Figure 7A(i)), with their bending axes oriented perpendicular to the direction of propulsion, corresponds to x‐axis in Figure 7A(ii). For untethered operation, the control board is mounted on a floating platform equipped with a buoyant unit. This design is inspired by underwater animals^[^ ^53^ ^]^ that use paired, symmetric fins to generate thrust while maintaining stability and enabling precise directional control. The fins operate at a submerged depth of 10 cm below the water surface (z‐direction).

The performance of the untethered swimming robot was evaluated under different base excitation controllers explained in Sections 2.3 and 4. Six different base excitation controllers, c1‐c6 explained in Section 2.3 were tested to evaluate the robot's performance. Free‐swimming experiments were performed in a 3‐meter‐long pool starting from the rest. Resultant swimming trajectories for six different control signals (c1 to c6) are shown in Figure 7A(iii), where each dot marks the robot's position at 5‐s intervals over a 25‐s duration. Measured average swimming speeds for controllers c1 through c6 were 0.11, 0.10, 0.07, 0.02, 0.07, and 0.05 m s^−1^, respectively. Controllers with faster flapping, where the period of base excitation is shorter yielded higher velocities (c1, c2 > c3, c5, c6 > c4). Furthermore, amplitude of stroke also affected swimming speed, with c5 outperforming c3 and c6 despite having identical stroke periods. Notably, controllers with symmetric durations for the power and return phases achieved slightly higher speeds (c1 > c2), which contrasts with conventional flapping fin dynamics where a faster power stroke generally increases velocity. This difference arises because rapid drag strokes lead to incomplete fin deployment during the power stroke; thus, maximizing asymmetry requires ensuring a full range of motion during the transition between power and return phases. These findings suggest that swimming performance is governed by speed‐ and configuration‐ dependent drag characteristics, highlighting the importance of anisotropic hydrodynamic effects on robot locomotion. Also, the additional comparison between the langoustine fin and other anisotropic fins is presented in Figure S7 (Supporting Information) to highlight the role of the langoustine fin in swimming.

A second robotic platform is a single anisotropic finger manipulator mounted on a robotic arm demonstrated effective object handling capabilities (Figure 7B). The manipulator successfully drags and reorients objects spread across a flat table toward a designated target region marked as orange tape(in Movie S2, Supporting Information). Through the coordinated use of both linear and rotational stiffening mechanisms, it adapts its compliance to the task—engaging higher stiffness for controlled pushing for dragging object and lower stiffness for passive deformation when passing through the gaps in between objects. This adaptability enables the system to navigate narrow or constrained regions in a compliant state, while retaining sufficient rigidity for effective interaction with the environment. On the plane, the manipulator handled objects of diverse shapes, sizes, and materials—including items such as a highlighter pen (20 g), AirPods (62 g), and tape rolls (43 g)—with weights (20, 50, 100, and 500 g) by dragging them with 3 g of exoskeleton manipulator.

The final utilization of the bio‐hybrid bending actuator is as a gripper equipped with two tendon‐augmented exoskeletons (Figure 7C(i)) and in Movie S4, Supporting Information), demonstrating the ability to grasp objects of various sizes and weights: a 19 g tomato replica (5.0 cm height, ≈7.2 cm diameter); a replica of a cherry‐tomato bundle (total weight 31 g) with each cherry tomato measuring 3.5 cm in height and 2.2 cm in diameter; a replica of a single cherry tomato (5 g) with a spherical shape and radius of 1.2 cm; a 20 g highlighter pen (1.2 cm × 2 cm cross‐section, 8 cm length); 62 g AirPods case (6 cm × 4.5 cm × 2 cm width × height × depth); and a wooden block (15 g, 1.5 cm × 3 cm × 5 cm). The grasping performance is strongly influenced by tendon routing, which governs the deformation and conformation of the exoskeleton. By selectively adjusting the tendon paths (Figure 7C(ii)), comparing route 1 and route 2, different grasping configurations are achieved. Route 1 is well suited for an enveloping grasp, effectively wrapping around the object. Route 2 is better for pinch grasping, allowing the gripper to hold objects with small grasp contacts and to support heavier objects than route 1. This enables the gripper to realize multiple grasping modes, conforming to irregular geometries and establishing stable contact with a diverse range of objects.

Throughout the robot testing process, biodegradable robot components—such as the exoskeleton and small synthetic materials used for assembly (e.g., cable ties, tendons wire, and elastomers) were disposed of after each use, but the actuators were retrieved and reused across multiple trials. This demonstrates a circular design approach to design and experimentation, introduced in Section 1, in which key functional elements are preserved and reused, while only minimal and biodegradable parts are replaced as needed.

## 3 Conclusion

This study proposed a new design approach for versatile biohybrid robots using the remains of dead animals' exoskeletons. Starting from an investigation of the natural mechanical features of the langoustine exoskeleton, we integrated synthetic components to demonstrate that such bio‐derived structures can be implemented to robotic mechanisms, from manipulators to locomotors. By utilizing inanimate exoskeletons integrated with synthetic components, we demonstrated that useful mechanical property can be generated such as directional force, or diverse motion. In particular, with tendon augmentation by varying the routing of the driving tendon within the same structure enabled the creation of multiple motion patterns under underactuation, allowing for object interaction, grasping, and manipulation. To support this, we developed a tendon routing model that facilitates understanding of the system kinematics and can serve as a foundation for analyzing and scaling future exoskeletal designs. Table S1 (Supporting Information)  presents a comprehensive comparison between existing necrobotic studies and our approach. A common limitation of previous works lies in their restricted working hours, typically lasting only a few hours to a few days, and occasionally up to 30 days in the case of fungi.^[^ ^25^ ^]^ In this work, we introduced barrier coating to extend the functional duration of necrobotic systems, including the restoration of functionality by water supply. Another limitation is the fixed design space inherent to natural body morphologies, which is determined by the original biological form. By employing different actuation methods, we proposed several robotic applications utilizing the langoustine exoskeleton, ranging from grasping to locomotion. Furthermore, by underactuating a continuum exoskeleton through tendons with varied routing, we explored morphological variations within the working space. These approaches are well suited for application in future necrobotic research.

Due to the inherent nature of biological organisms, individual specimens exhibit morphological variations that arise from their biological origin. This natural variability imposes limitations on the precision of structural design and simulation. Overcoming these constraints will require the intelligent design of synthetic mechanisms and the development of tunable controllers. Furthermore, detailed structural characterization and precise experimental measurements will be critical to maximizing their utility. Continuous advancements in durability and robustness will also be necessary to enable their use as more functional robotic architectures. Another key challenge lies in integrating these naturally variable structures into systems requiring high repeatability, which will demand hybrid approaches combining bio‐derived components with precision‐fabricated synthetic parts.^[^ ^54^ ^]^

Langoustine bio‐exoskeletons are exceptionally lightweight yet durable and robust, capable of withstanding substantial forces due to their role as natural armor. While they are not rapidly biodegradable, their structural motifs are conserved across many biological species, making them promising candidates for scalable and tunable machine designs. Moreover, such biotic exoskeletons represent a promising class of mechanisms for future applications aligned with recent advances in material‐driven actuation systems.^[^ ^54^ ^]^ To take full advantage of this biological repertoire of exoskeletons, a deeper understanding of joint mechanics, structural properties, and compatible synthetic actuation mechanisms is essential.

Notably, the bio‐exoskeletons collected in this study were sourced from food waste, demonstrating the potential for sustainable robotic design^[^ ^40^ ^]^ through the repurposing of biological materials. These materials are biocompatible and have minimal environmental impact during use and disposal, and have a core element for sustainable circular design.^[^ ^55^ ^]^ Although in the presented form only the exoskeletal mechanism utilized biotic materials, there are many promising directions for expanding the use of nature‐based materials, such as fiber‐based structures for tendon‐driven systems,^[^ ^56^, ^57^, ^58^ ^]^ as well as their combination with bioactuators^[^ ^59^ ^]^ toward fully biotic robots.

To quantify the effect of cyclic design, sustainability metrics can be set such as embodied carbon, which is the total amount of carbon dioxide emitted throughout the entire lifecycle. For different swimming fins explained in Figure S7 (Supporting Information), we can make a comparison. Table S2 (Supporting Information) summarizes the environmental indicators of each fin, including material composition, embodied CO_2_, and biodegradability. It can be observed that fin 2 (TPU) shows the highest embodied carbon due to its petroleum origin and low biodegradability, whereas fin 1 and fin 3, composed mainly of PLA, exhibit much lower embodied CO_2_. The langoustine fin consists of a chitin biopolymer that demonstrates both the lowest embodied CO_2_ ≈0.5 and full biodegradability, suggesting its strong potential for circular and sustainable design. Looking forward, such bio‐derived structural elements could be utilized for biomedical implants, bio‐system monitoring platforms, or the creation of artistic robotic installations that highlight the aesthetic qualities of biological architectures. Though nature does not necessarily provide the optimal form of functionality,^[^ ^60^ ^]^ it still outperforms many artificial systems, and offers valuable insights for designing functional machines based on elegant principles.

## 4 Experimental Section

### Repurposing Process and Fabrication

### Repurposing

To repurpose the exoskeleton, commercially frozen whole langoustines (Læsø Choice), flash‐frozen at –18°C, were first fully thawed at room temperature. The abdomen sections were then separated from the cephalothorax, and immersed in boiling water for 2 min for cooking. After boiling, the inner flesh was carefully removed by tweezers, and the remaining exoskeletal shells were rinsed.

### Storage

The processed exoskeleton samples were placed in airtight food‐grade snap‐lock container lined with moistened paper towels to maintain ambient humidity. The containers were then stored in a refrigerator at 5°C to prevent dehydration and material degradation. As storage time can affect the moisture level, samples for moisture‐sensitive experiments, such as the working‐hour measurement, were used immediately after thawing without additional storage.

### Augmentation

The rinsed exoskeleton was fixed in its maximum extension configuration. On the dorsal shell, the center of each tergite was punctured using a 0.5 mm diameter sewing needle. A 2 mm thermoplastic polyurethane (TPU) elastomer band (Daiso) was used as the spring element along the back and threaded through the punctured holes. Using a thin string (0.08 mm, 4‐braid line, EFFZETT), the elastomer was sewn to each tergite. At each sewing knot, a drop of cyanoacrylate adhesive (UHU Sekundenkleber) was applied to bond the TPU and reinforce the knot. This ensured that each segment acted as an independent spring, even though the elastomer was originally a continuous band.

After the adhesive had dried, the tendon was routed. At the anchor point, an inextensible string (0.22 mm, 8‐carrier superbraid fishing line, WFT) was tied to the shell, with the same adhesive applied to strengthen the fixation. Using the sewing needle, the tendon was then routed along the designed path, passing in and out of the shell.

### Coating

Samples were dip‐coated with silicone(Smooth‐On EcoFlex 00–50 and Dragon Skin 00–10) cured in room temperature for 4 h, the latter mixed with silicone fluid(Smooth‐on Silicone Thinner) 10 % mixing ratio by weight to reduce viscosity. Curing of the coating material was done with the abdomen exoskeleton in its fully extended configuration, fitted onto a rod of straw.

### Kinematic Model

To analyze the behavior of the scampi exoskeleton and simulate its deformation under tendon‐driven actuation, we construct a planar tendon‐driven linkage model that reproduces the articulated morphology of the abdomen. The model consists of seven serially connected rigid links: six jointed abdominal segments and one terminal tail link. Each abdominal segment is represented as a kite‐shaped rigid plate composed of left and right triangular elements, corresponding to the anatomical sternites and lateral plates. This triangular geometry encodes the anatomical anchor points for tendon routing.

The dorsal side of the structure incorporates an elastomer element whose stiffness is represented in the model as joint rotational stiffness. Each of the six joints (*k*_1_–*k*_6_) connects adjacent kite‐shaped links, with stiffness parameters and rest angles determined from morphological measurements. The seventh link corresponds to the tail fan and is modeled as a simple rigid extension without a joint.

Tendon routing is defined by selecting anchor points located at specific vertices of the triangular elements. The tendon path lies along the ventral side of the abdomen and passes through intermediate pulleys determined by these anchor points. Different motion patterns are generated by altering the sequence of pulley points in the routing definition.

The forward kinematics is computed in 2D by sequentially transforming the local coordinates of each triangular element into the global frame. For a given set of joint angles

(1) $$\theta =[\theta_{1},\theta_{2},\cdots ,\theta_{6}]$$

each triangle is rotated and translated using a rigid‐body transformation:

(2) $$p^{\text{global}}=R\left(\phi_{i}\right)\;p^{\text{local}}+t_{i}$$

where the absolute orientation ϕ_*i*_ of the *i*‐th segment is the cumulative sum of joint angles,

(3) $$\phi_{i}=\sum_{j=1}^{i}\theta_{j},\;i=1,\cdots ,6$$

and the 2D rotation matrix **R**(ϕ_*i*_) is defined as

(4) $$R\left(\phi_{i}\right)=\left[\begin{array}{cc} \cos\phi_{i} & -\sin\phi_{i}\\ \sin\phi_{i} & \cos\phi_{i} \end{array}\right]$$

The translation **t**_*i*_ corresponds to the position of the previous segment's endpoint.

This procedure preserves the geometric layout of each kite‐shaped segment while updating all tendon anchor positions.

In the tendon‐driven linkage optimization, several boundary conditions are imposed to ensure that the computed postures are both mechanically feasible and anatomically realistic. First, joint angles are constrained within physiologically plausible limits using lower and upper bounds (**lb** and **ub**), defined from morphological measurements of the scampi abdomen. The lower bounds and upper bounds are from measured data from characterization(see Section 2.2). Additionally, the tendon length constraint enforces that the Euclidean distance sum between consecutive pulley points matches the prescribed target length for each simulation step.

(5) $$L_{\text{tendon}}=\sum_{k=1}^{N}{∥r_{k}-r_{k-1}∥}_{2}$$

where ${\left\{r_{k}\right\}}_{k=0}^{N}$ are the pulley points in global coordinates. This tendon length constraint is nonlinear and depends on the current joint configuration via forward kinematics. In combination with the fitness function, these bounds ensure that the optimization yields postures that respect joint motion limits, preserve tendon routing geometry, and maintains physical realism in the simulation.

Tendon‐driven motion is simulated by prescribing a tendon shortening profile and solving for the joint angles that minimize the total elastic energy stored in the joints:

(6) $$E=\frac{1}{2}\sum_{i=1}^{6}k_{i}{\left(\theta_{i,0}-\theta_{i}\right)}^{2}+\sum_{i=1}^{6}f_{i}\left(\theta_{i,0}-\theta_{i}\right)$$

subject to the constraint that the computed tendon length matches the target value. Here, *k*_*i*_ is the rotational stiffness, *f*_*i*_ is an optional bias torque term, θ_*i*, 0_ is the rest (upper‐bound at maximum extension) angle, and θ_*i*_ is the current joint angle.

The constrained optimization is solved in MATLAB using fmincon with a Sequential Quadratic Programming (SQP) algorithm. By iterating over incremental tendon pulls, the simulation produces a posture evolution sequence for the given tendon routing. This framework allows systematic exploration of tendon routing effects on motion and the relationship between posture and stored elastic energy in a tendon‐driven decapod's abdomen exoskeleton, which is analogous to continuum linkage structure.

### Experimental Setups

### Mechanical Strength Evaluation

The mechanical strength of the langoustine's abdomen exoskeleton was evaluated by measuring the maximum withstand force at the tip in at its maximum extension. The loadcell mounted at the tip of the robot arm(UR 5, Universal Robots) and pushes the tip of the exoskeleton in its extension direction and measured peak force. This was measured across five specimens with body weights ≈45 g and abdomens ≈8 cm long, and the average and standard deviation values were calculated.

### Anisotropy Measurement

Figure 3D illustrates this asymmetric stiffness profile. The abdomen undergoes a circular‐like trajectory (approximated with a moment arm of 60 mm) during flexion and extension, and torque measurements were recorded along this path. In addition to the range of motion, the stiffness of the bio‐jointed tail is important, to capture the required force required to actuate the joints. The stiffness was measured at the tail for both folding and unfolding, by measuring the force at the tail for different angles of curvature.

### Range of Motion Measurement

To assess the range of motion (RoM) across the six abdominal exoskeleton (J1–J6), we analyzed five langoustine exoskeletons with a similar size (net abdominal section weight ≈45 g) to account for natural variation in size, scale, and joint angles.

### Tendon Force‐Response Measurement

In Section 2.4.2, tendon force response was presented. A load cell was mounted on the UR5 robotic arm to measure tendon forces during controlled tendon displacement. The tendon was linearly pulled by 3 cm (with a pulling speed of 0.25 cm s^−1^) from the extended to the flexed position, during which the force profile was recorded. At the maximum flexion point, the tendon was then gradually released in the opposite direction while continuing to measure the tendon force. his procedure was repeated five times, and the resulting force data were averaged to obtain the mean force profile, with standard deviations calculated to assess measurement variability. This protocol allowed characterization of the force response during both tendon extension and relaxation phases.

### Force Response of Barrier‐Coated Exoskeletons

To demonstrate the effect of coatings, the force response during tendon pulling was measured for four cases: augmented without an coating (S1), augmented with EF 00–50 (S2), augmented with DS 00–10 (S3), and augmented without elastomer but with DS 00–10 (S4), as shown in Figure 6B.

### Working Hour Measurements

The working time of the abdomen joint was evaluated by manually pulling the tendon attached to the joint every hour until mechanical failure occurred. In one condition, the joint was left uncoated. In another condition, the joint was wrapped with a wet tissue to supply water for 1 h at a time. This water supplementation was applied twice during the test period. Additionally, joints coated with EF and DS coatings were tested under the same conditions to assess their effect on durability.

### High‐Speed Actuation

For rapid cyclic actuation involving quick tendon pulling and release, the tendon was connected to a crank mechanism driven by a motor with a free‐load speed of ≈600 RPM. To ensure reliable operation, the crank mechanism and the exoskeleton were firmly mounted. As bonding with adhesive proved insufficient to secure the assembly, manual fixation was employed to achieve the required mechanical robustness.

### Robotic Platforms

### Swimming Robot

The untethered swimming robot is equipped with two exoskeleton‐inspired fins, each actuated at the base by a waterproof servo motor (Dynamixel XW430‐T200‐R) connected with a 3D‐printed fin holder. The motors are driven through a U2D2 serial connection and controlled by a Raspberry Pi microcontroller via Wi‐Fi. The entire system is mounted on a floating platform composed of swimming foam blocks as buoyant unit, which support the plate with a microcontroller, a motor power supply (U2D2 PHB Set), and a 10 000 mAh power bank for untethered operation.

Fin motion follows a triangular wave, as observed in underwater animals,^[^ ^61^ ^]^ characterized by amplitude *A*, period *T*, and starting angle ϕ_min_. The control signal is modulated by a duty ratio—the ratio of the power stroke duration to the total cycle—resulting in either symmetric or asymmetric motion. The power stroke lasts for a duration of *T*_*c*_, followed by a return stroke of duration *T*_*o*_. The velocity ratio α = *T*_*c*_/*T* defines the fraction of the cycle spent in the power stroke. The amplitude *A* is set to half the maximum fin angle (ϕ_max _/2). By adjusting *A*, *T*, and θ_max _, different propulsion patterns can be generated.

Let the normalized time be $t_{\mathrm{mod}}=t\;\mathrm{mod}\;T$. The fin angle ϕ(*t*) over one cycle is defined as:

(7) $$\phi \left(t\right)=\left\{\begin{array}{cc} \phi_{\max}-\left(\phi_{\max}-\phi_{\min}\right)\frac{t_{\mathrm{mod}}}{T_{c}}, & 0\leq t_{\mathrm{mod}}<T_{c}\\ \phi_{\min}+\left(\phi_{\max}-\phi_{\min}\right)\frac{t_{\mathrm{mod}}-T_{c}}{T-T_{c}}, & T_{c}\leq t_{\mathrm{mod}}<T \end{array}\right.$$

where $t_{\mathrm{mod}}=t\;\mathrm{mod}\;T$.

During the power stroke phase (0 ⩽ *t*_mod_ < *T*_*c*_), the fin sweeps from ϕ_max _ to ϕ_min _, generating thrust. During the return stroke phase (*T*_*c*_ ⩽ *t*_mod_ < *T*), the fin moves back from ϕ_min _ to ϕ_max _, completing the propulsion cycle (Table 1).

**Table 1:** Controller parameters for base excitation.

Controller	*T* [s]	Duty ratio	ϕ_min _‐ϕ_max _ [deg]
C1	1	0.5	0–150
C2	1	0.3	0–150
C3	2	0.5	0–150
C4	4	0.5	0–150
C5	2	0.5	0–175
C6	2	0.5	15–135

### Data Collection

### Imaging and Imaging Analysis

Robot movies were recorded using an iPhone 15 Pro (30 fps for normal speed and 240 fps for slow motion), and a GoPro for underwater swimming. For the high‐speed movement a Photron FASTCAM Nova S16 high‐speed camera (2000 fps, played back at 0.01× real‐time) was used, and for a microscopic image for langoustine components, we used is called optical microscope, Nikon Eclipse TS100. We examined the samples under magnifications 10× and 20×. For image‐based tracking, Tracker Video Analysis and Modeling Tool (Tracker Video Analysis and Modeling Tool for Physics Education. https://www.physlets.org/tracker/)

## Conflict of Interest

The authors declare no conflict of interest.

## Supporting information



Supporting Information

Supplemental Movie 1

Supplemental Movie 2

Supplemental Movie 3

Supplemental Movie 4

## Data Availability

The data that support the findings of this study are available from the corresponding author upon reasonable request.
