# Supplementary material for: Dead Matter, Living Machines: Repurposing Crustaceans' Abdomen Exoskeleton for Bio‐Hybrid Robots
Source: Adv Sci (Weinh). 2025 Nov 26;13(15):e17712. doi: 10.1002/advs.202517712 (PMC13042951; doi:10.1002/advs.202517712)
Supplement: Supplementary file 1 — Supporting Information [file ADVS-13-e17712-s002.pdf]

Supporting information

Dead Matter, Living Machines: Repurposing Crustaceans' Abdomen Exoskeleton for Biohybrid Robots

*Sareum Kim, Kieran Gilday, and Josie Hughes**

Affiliations:
All authors are with the CREATE Lab, Institute of Mechanical Engineering, Swiss Federal Institute of Technology in Lausanne (EPFL), 1015 Lausanne, Switzerland.
josie.hughes@epfl.ch

The PDF file includes:
Supplementary Text
Figure S1 to S5
Figure S1. Repurposing process of langoustine exoskeleton from food to food-waste.
Figure S2. Structure of the langoustine exoskeleton, cross-sectional view after splitting into left and right halves along the dorsal line.
Figure S3. Material properties of langoustine biotic materials before and after cooking.
Figure S4. Kinematic simulation of the tendon-driven exoskeleton with embedded elastomer, showing motion from the extended state to the flexed state as the tendon is pulled.
Figure S5. Untethered swimming robot platform.
Figure S6. Tip trajectories of the exoskeletal flapping fin under different base excitation controllers (C1 to C6), sampled at every 1/8 of the base excitation period.

Supplementary Movies:
Movies S1 to S4
Supplementary Movie S1. Directional thrust generation by base excitation.
Supplementary Movie S2. Object handling with exoskeletal manipulator.
Supplementary Movie S3. High-speed bending with tendon-augmented exoskeleton.
Supplementary Movie S4. Object grasping with tendon-driven exoskeletal gripper.

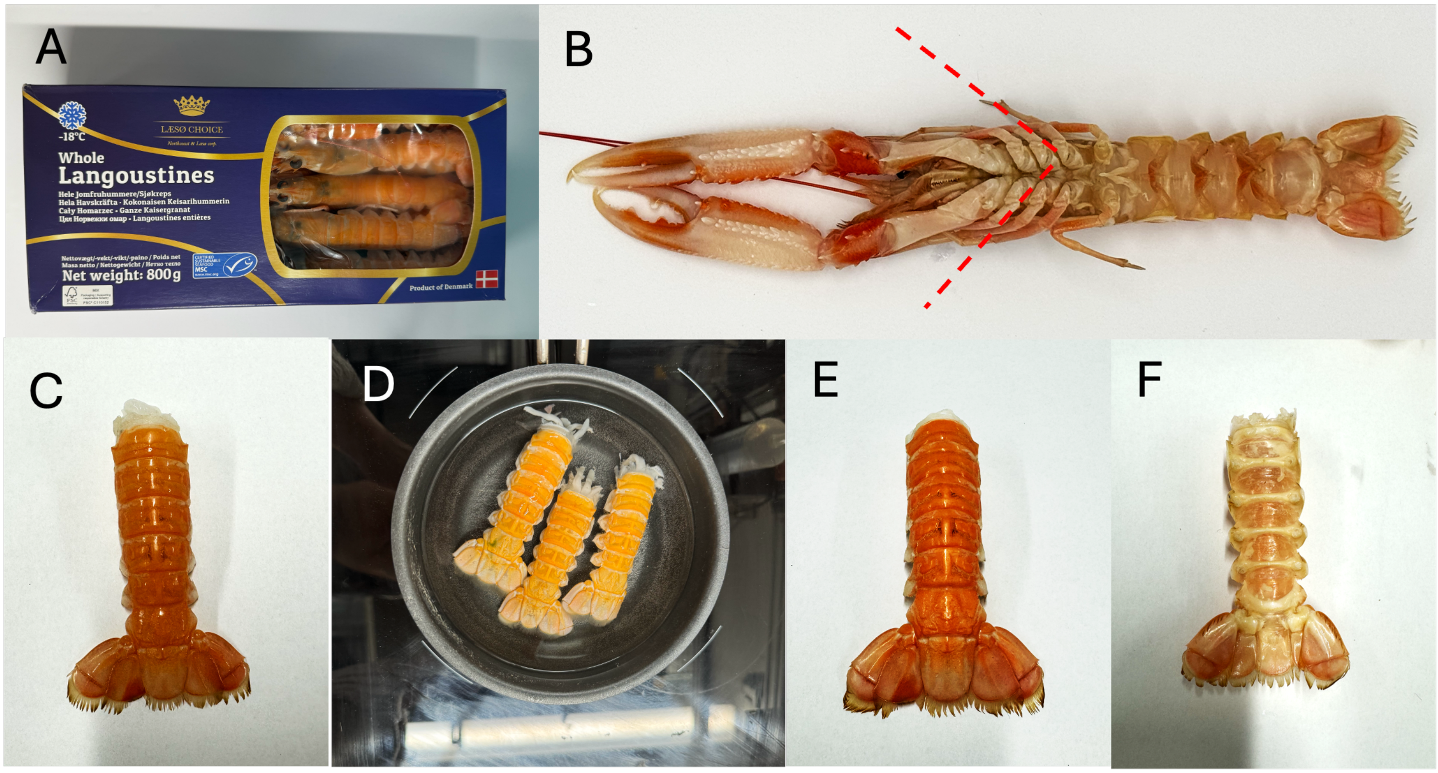

Figure S1. Repurposing process of langoustine exoskeleton from food to food waste. (A) Frozen langoustine from a grocery store, thawed at room temperature (B) the carapace (body) and abdomen are separated by cutting along the V-shaped dotted lines (C) separated abdomen with muscle tissue (D) Abdomen cooked in boiling water for 2 minutes (E) cooked abdomen with the shell becoming opaque (F) meat removed with tweezers, leaving an empty exoskeleton.

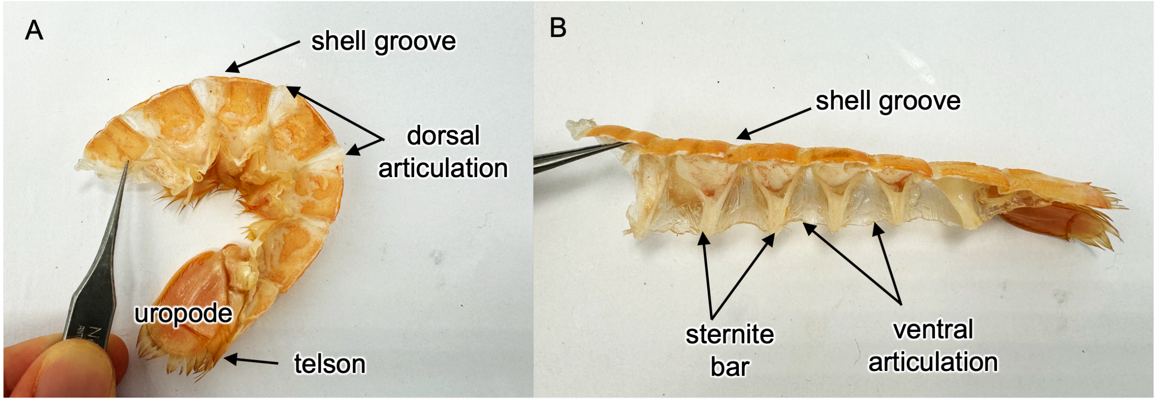

Figure S2. Material properties of langoustine biotic materials before and after cooking. The meat underwent significant changes during cooking, transforming from a soft, transparent tissue into a tough, elastic, and opaque white mass. The shell also became more opaque after cooking. Notably, the dorsal and ventral articulations retained their mechanical properties, and the structural grain of the membrane remained preserved.

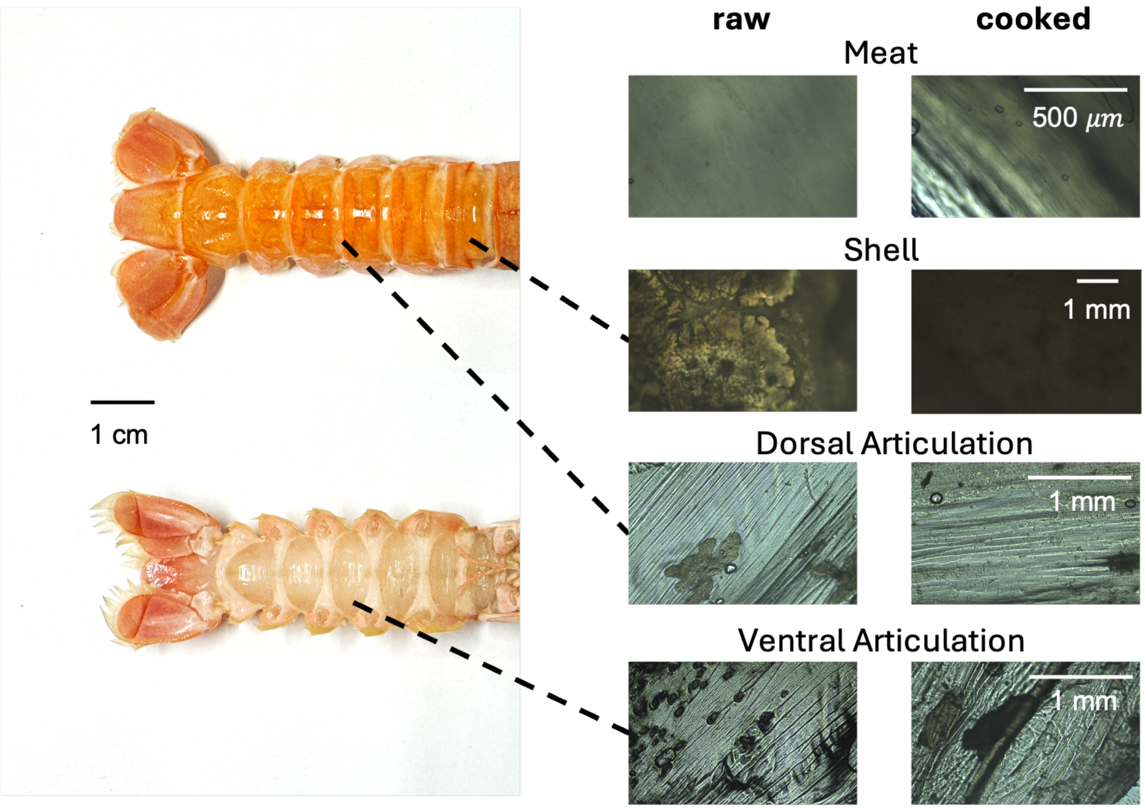

Figure S3. Structure of the langoustine exoskeleton, cross-sectional view after splitting into left and right halves along the dorsal line. (A) Flexed state, highlighting the maximum stretch of the dorsal articulation (B) extended state, where the ventral articulation is maximally stretched and the shell groove contacts the neighboring segment.

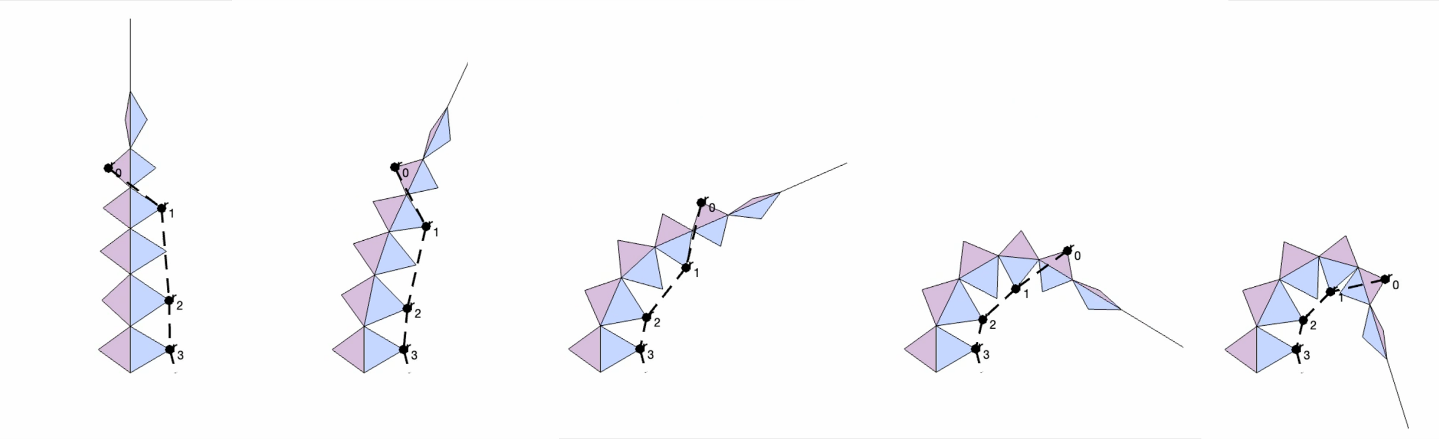

Figure S4. Kinematic simulation of the tendon-driven exoskeleton with embedded elastomer, showing motion from the extended state to the flexed state as the tendon is pulled.

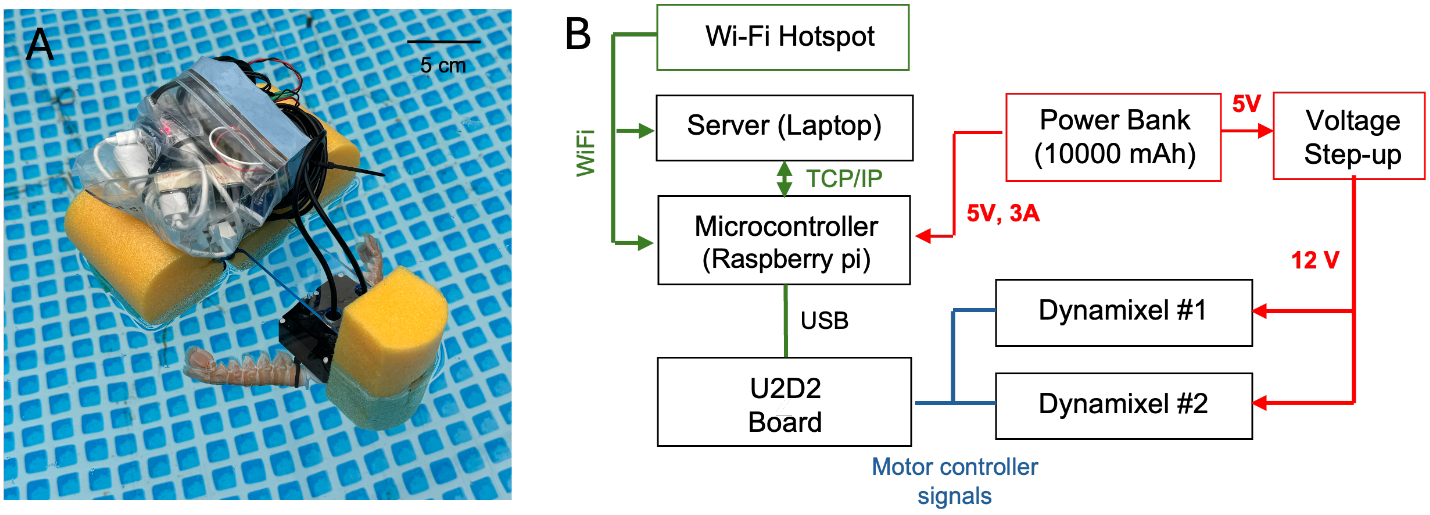

Figure S5. Untethered swimming robot platform. (A) The swimming robot floating on water (B) schematic diagram of the robot's electronics, communication, and power systems.

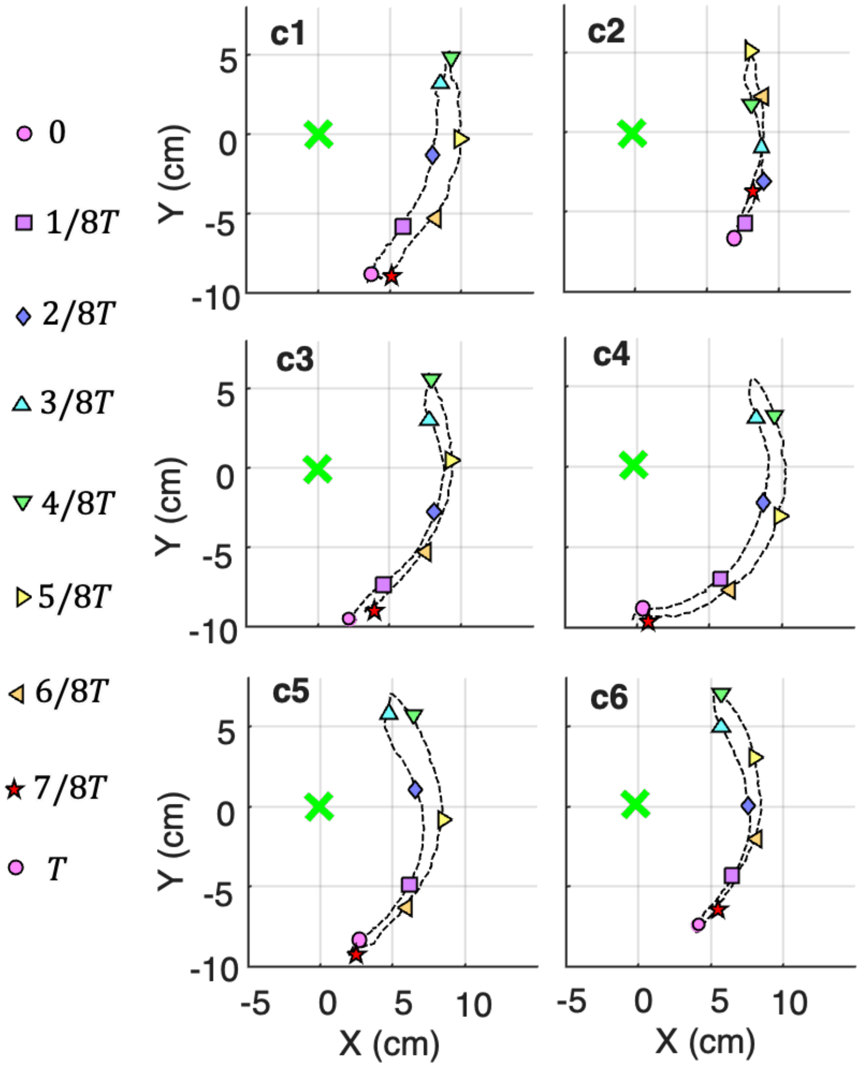

Figure S6. Tip trajectories of the exoskeletal flapping fin under different base excitation controllers (C1 to C6), sampled at every 1/8 of the base excitation period. The base position is marked by a green '×' at the origin (0, 0).

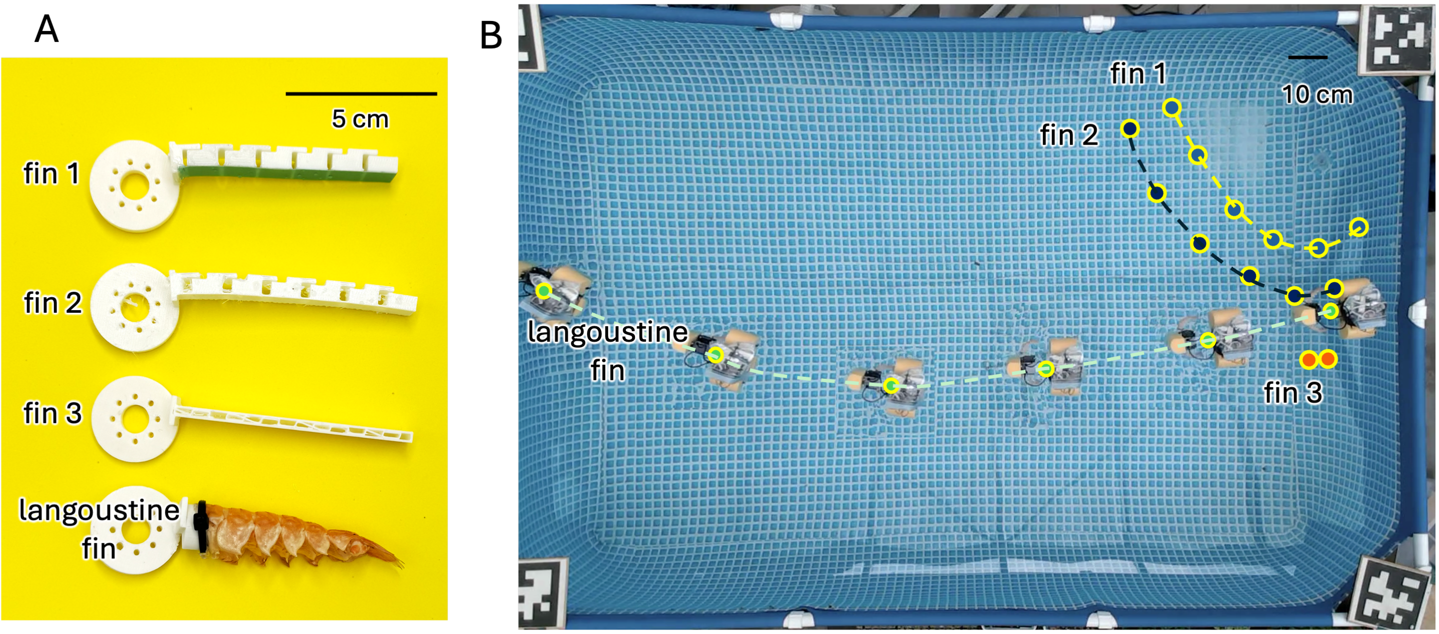

Figure S7. Free-swimming trajectories of the robot under flapping controller c1, with different fin designs in similar length with langoustine fin, (A) different fin designs, fin 1: segmented PLA fin with flexible PP backbone, fin 2: segmented TPU fin, and fin 3: PLA paddle fin, and langoustine fin, (B) trajectories of 4 different fins, where each dot represents a 5-second interval.

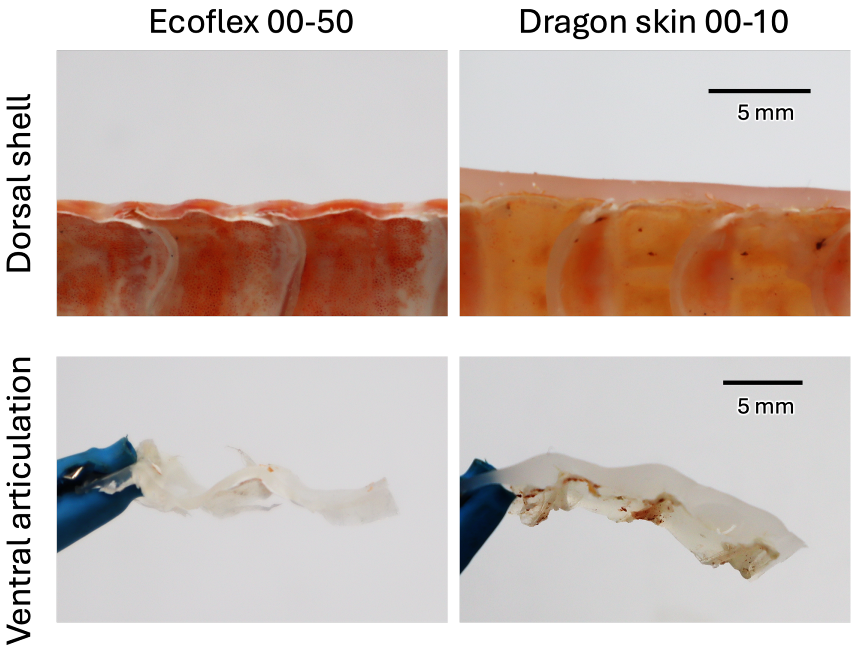

Figure S8. Cross-sectional view of the barrier coating on the langoustine exoskeleton with two silicone materials (Ecoflex 00-50 and Dragon Skin 00-10) applied to the dorsal shell and the ventral articulation.

Work	Organism	Biological Part Used	Function/Actuator	Size	Duration	Performance Metrics
[S1]	wolf spider (*Lycosidae*)	Whole body (legs)	Gripper /pneumatics	Body 25 mm; leg 45–65 mm; 33.5 mg	∼2 days	Max force 0.35 mN; load ratio 1.3×; response <1 s
[S2]	five-horned beetle (*Eupatorus gracilicornis*)	Exoskeleton (whole)	Walking robot / motors	58.2 mm body length, 7.3 g weight	Multi-day stable operation	Payload ratio 6847% (500 g); speed 1.55 mm/s; specific power 0.28 W/kg
[S3]	hawkmoth (*Manduca sexta*)	Antennae	Odor sensor / n/a	Drone ∼85 mm; 30 g	2–4 h	Response 0.045 s; power 2.7 mW
[S4]	silkworm moth (*Bombyx mori*)	Antennae	Odor sensor / n/a	sensor 15×23 mm (15.5 g)	4.5 h (EAG stable)	Detection limit 1 pg; search range 5 m; success 41%; power 0.61–0.88 W
[S5]	king oyster mushroom (*Pleurotus eryngii*)*	bioelectrical controller	Bioelectrical controller / n/a	n/a	Activity ≥30 days	Spikes 135 µV avg (1.8 mV max); response 1–2 s
[S6]	Asian water monitor (*Varanus salvator*)	Complete skeleton	Quadrupedal necrobot / motors	∼2 m length	n/a	Gait similarity 91.7%
This work	Langoustine (*Nephrops norvegicus*)	abdomen skeleton	Gripper, manipulator, and swimming fin / motors	∼ 10 cm length, ∼ 3 g	up to 38 hours	blocked force 660 gf; fast bending 8 Hx; dragging 500 g; swimming speed 0.11 m/s

n/a : Not Available
* Living tissue

Supplementary Table S1. Comparison in between necrobotic systems.

Type	Material breakdown (g)	Embodied CO_2 (g)	Biodegradability
Segmented PLA fin with flexible PP backbone (fin1)	PLA* 3.0 , PP** 0.1	1.6	high for PLA, low for PP
Segmented TPU fin (fin2)	TPU*** 3.3	8.7	Low
PLA paddle fin (fin3)	PLA* 2.6	1.3	High
Langoustine fin	Chitin/chitosan 3.0	0 †	Fully degradable

* The embodied CO_2 value for PLA was obtained from [S7]
** The embodied CO_2 value for PP was obtained from [S8]
*** An average embodied CO_2 of 2.8 kg CO_2-eq kg^{-1} for PU was adopted from [S9]
† Material derived from food waste are considered carbon-neutral, as no additional production processes are involved [S10]

Supplementary Table S2. Comparative sustainability assessment of swimming fins.

References

[S1] T. F. Yap, Z. Liu, A. Rajappan, T. J. Shimokusu, D. J. Preston, *Advanced Science* **2022**, *9*, 29 2201174.

[S2] Y. Tsvetkov, P. Alam, *bioRxiv* **2024**, 2024–11.

[S3] M. J. Anderson, J. G. Sullivan, T. K. Horiuchi, S. B. Fuller, T. L. Daniel, *Bioinspiration & Biomimetics* **2020**, *16*, 2 026002.

[S4] C. Fukui, T. Uchida, S. Koizumi, Y. Murayama, H. Liu, T. Nakata, D. Terutsuki, *npj Robotics* **2025**, *3*, 1 4.

[S5] A. K. Mishra, J. Kim, H. Baghdadi, B. R. Johnson, K. T. Hodge, R. F. Shepherd, *Science Robotics* **2024**, *9*, 93 eadk8019.

[S6] L. Foulds, D. S. Yudha, P. Alam, *bioRxiv* **2025**, 2025–07.

[S7] E. Rezvani Ghomi, F. Khosravi, A. Saedi Ardahaei, Y. Dai, R. E. Neisiany, F. Foroughi, M. Wu, O. Das, S. Ramakrishna, *Polymers* **2021**, *13*, 11 1854.

[S8] A. Alsabri, F. Tahir, S. G. Al-Ghamdi, *Polymers* **2021**, *13*, 21 3793.

[S9] N. Von der Assen, A. Bardow, *Green Chemistry* **2014**, *16*, 6 3272.

[S10] C. Lopes, L. T. Antelo, A. Franco-Uría, A. A. Alonso, R. Pérez-Martín, *Journal of Cleaner Production* **2018**, *172* 4140.
